# Identification of glutamine as a potential therapeutic target in dry eye disease

**DOI:** 10.1038/s41392-024-02119-1

**Published:** 2025-01-22

**Authors:** Xiaoniao Chen, Chuyue Zhang, Fei Peng, Lingling Wu, Deyi Zhuo, Liqiang Wang, Min Zhang, Zhaohui Li, Lei Tian, Ying Jie, Yifei Huang, Xinji Yang, Xiaoqi Li, Fengyang Lei, Yu Cheng

**Affiliations:** 1 Department of Ophthalmology, the Third Medical Center of Chinese PLA General Hospital, Beijing, China; 2 Department of Nephrology, the First Medical Center of Chinese PLA General Hospital, State Key Laboratory of Kidney Diseases, Beijing, China; 3 Beijing Institute of Ophthalmology, Beijing TongRen Eye Center, Beijing Key Laboratory of Ophthalmology and Visual Sciences, Beijing Tongren Hospital, Capital Medical University, Beijing, China

**Keywords:** Drug development, Therapeutics, Immunological disorders

## Abstract

Dry eye disease (DED) is a prevalent inflammatory condition significantly impacting quality of life, yet lacks effective pharmacological therapies. Herein, we proposed a novel approach to modulate the inflammation through metabolic remodeling, thus promoting dry eye recovery. Our study demonstrated that co-treatment with mesenchymal stem cells (MSCs) and thymosin beta-4 (Tβ4) yielded the best therapeutic outcome against dry eye, surpassing monotherapy outcomes. In situ metabolomics through matrix-assisted laser desorption/ionization mass spectrometry imaging (MALDI-MSI) revealed increased glutamine levels in cornea following MSC + Tβ4 combined therapy. Inhibition of glutamine reversed the anti-inflammatory, anti-apoptotic, and homeostasis-preserving effects observed with combined therapy, highlighting the critical role of glutamine in dry eye therapy. Clinical cases and rodent model showed elevated expression of glutaminase (GLS1), an upstream enzyme in glutamine metabolism, following dry eye injury. Mechanistic studies indicated that overexpression and inhibition of GLS1 counteracted and enhanced, respectively, the anti-inflammatory effects of combined therapy, underscoring GLS1’s pivotal role in regulating glutamine metabolism. Furthermore, single-cell sequencing revealed a distinct subset of pro-inflammatory and pro-fibrotic corneal epithelial cells in the dry eye model, while glutamine treatment downregulated those subclusters, thereby reducing their inflammatory cytokine secretion. In summary, glutamine effectively ameliorated inflammation and the occurrence of apoptosis by downregulating the pro-inflammatory and pro-fibrotic corneal epithelial cells subclusters and the related IκBα/NF-κB signaling. The present study suggests that glutamine metabolism plays a critical, previously unrecognized role in DED and proposes an attractive strategy to enhance glutamine metabolism by inhibiting the enzyme GLS1 and thus alleviating inflammation-driven DED progression.

## Introduction

The aging population and increased screen usage among youth have elevated the prevalence of dry eye disease (DED). DED is primarily characterized by an imbalance in tear film homeostasis, accompanied by ocular discomfort and/or ocular surface histopathological features^1^. Globally, DED’s prevalence spans from approximately 5% to 50%^2^. In clinical practice, DED stands as the second most prevalent ocular disease, trailing only refractive error. It significantly impacts patients’ quality of life, and if untreated, DED can result in sustained damage to the delicate cellular layer of the ocular surface^3^. Nevertheless, current treatment options primarily offer palliative relief, including artificial tears, nutritional supplements, and topical steroids^1^, with no definitive cure in sight. Therefore, the development of innovative alternative therapies for DED holds paramount importance.

DED is an adaptive immune-mediated inflammatory condition, with metabolic pathways emerging as crucial regulators of inflammatory responses^4,5^. In the context of inflammation, epithelial and immune cells shift their energy supply from oxidative phosphorylation to glycolysis^6–8^. Additionally, maintaining cell membrane stability via the glycerophospholipid pathway assumes importance, and deviations in glycerophospholipid metabolism within the cornea post DED can potentially trigger inflammatory responses^9^. Heightened sphingomyelin metabolism alteration in the cornea also contributes to inflammation, cell death, and corneal damage^10^. Furthermore, altered amino acid metabolites like arginine, phenylalanine, and betaine are reduced in tear fluid of severe ocular surface disease patients, possibly linked to ocular surface inflammation^9,11^. Such metabolic remodeling collectively influences corneal internal environment and nutritional milieu, exerting a pivotal role in regulating inflammatory responses. The academic consensus suggests that the pathology of DED primarily affects the lacrimal functional unit, with corneal epithelial cells being the most significantly damaged cell type. Recent studies indicate that epithelial cell injury across various organs is associated with metabolic changes^6–8^. Here, we proposed a hypothesis that the damage to cornea caused by dry eye may occur through metabolic pathways, and we aimed to identify the key target metabolites involved in this process. To investigate this, we employed high-resolution mass spectrometry tissue imaging via matrix-assisted laser desorption ionization mass spectrometry imaging (MALDI-MSI) and metabolism analysis techniques using a dry eye animal model. In the last decade, metabolomics applications in dry eye have predominantly centered on tears^12–15^ and serum^16^ analyses, with limited focus on in-situ metabolomics of ocular tissues in the context of dry eye. The emergence of MALDI-MSI technology^17^ presents a novel avenue for in situ visualization. In contrast to conventional metabolomics, which merely offers semiquantitative data regarding metabolite abundance, devoid of spatial distribution insight, MALDI-MSI enables label-free quantification of metabolites directly within tissue sections. This innovative technique enabled direct in-situ metabolite assessment and visualization in tissue sections as was indicated by our preceding research^17^. Our findings confirmed that metabolic alterations in corneal play a crucial role in DED.

Mesenchymal stem cells (MSCs) have been recognized as a promising strategy for ocular regenerative repair^18^.Investigations have suggested the efficacy of MSCs across various ocular diseases, including dry eye, corneal epithelial injury, and ischemic optic nerve injury^19,20^. Reports demonstrate that MSCs exhibit the capacity to reinstate mitochondrial function and augment oxidative phosphorylation through processes encompassing mitochondrial translocation, extracellular vesicle secretion, and release of cell growth factors^21,22^. These mechanisms subsequently contribute to the regulation of cellular metabolism^23^. Research indicates that MSCs can improve metabolic alterations in aged rats by modulating metabolic dynamics, thereby enhancing liver regeneration and preventing age-related decline^24^. Furthermore, MSCs exert immunoregulatory effects by altering the metabolism of immune cells^25^. Thymosin beta-4 (Tβ4) is a 43-peptide molecule with N-terminal acetylation, which effectively preserves goblet cell density in conjunctival tissue and mitigates corneal epithelial peeling associated with DED^26^. According to reports, MSCs possess remarkable self-renewal and differentiation potential, allowing them to differentiate into epithelial-like cells and facilitate corneal regeneration^22,27^. On the other hand, Tβ4 enhances the migratory capacity of cells across various tissues, thereby promoting the migration of healthy corneal epithelial cells^26^. Furthermore, Tβ4 can promote the proliferation of MSCs and enhance their effective migration to injury sites^28^, thus improving corneal regenerative repair. The combined application of Tβ4 and MSCs strengthens the anti-apoptosis, ant-inflammation and metabolism modulation, ultimately enhancing the regeneration and repair of the cornea.

In this study, we introduced a novel approach involving Tβ4 combined with MSCs (MSC + Tβ4) for dry eye treatment and identified glutamine as a potential therapeutic target in dry eye disease. We introduced a novel methodology utilizing MALDI-MSI for in situ metabolomics. This innovative molecular imaging technique integrates high-throughput mass spectrometry analysis with two-dimensional spatial imaging, enabling the simultaneous detection of multiple substance types while allowing for relative quantification and spatial distribution analysis. Our study investigated the metabolic changes of DED, achieving a visual assessment of metabolites within ocular tissues. Notably, we identified for the first time the regulatory role of glutamine metabolism in modulating dry eye inflammation and confirmed its critical function during the progression of dry eye disease. This finding presents a new therapeutic target that addresses existing gaps in the understanding of DED treatment, thereby offering a novel strategy for the clinical application of glutamine in the management of dry eye disease.

## Results

### Combining MSC and Tβ4 therapy improves dry eye, leading to enhanced restoration of tear secretion, amelioration of pathological damage, and reduced apoptosis

**Fig. 1:**
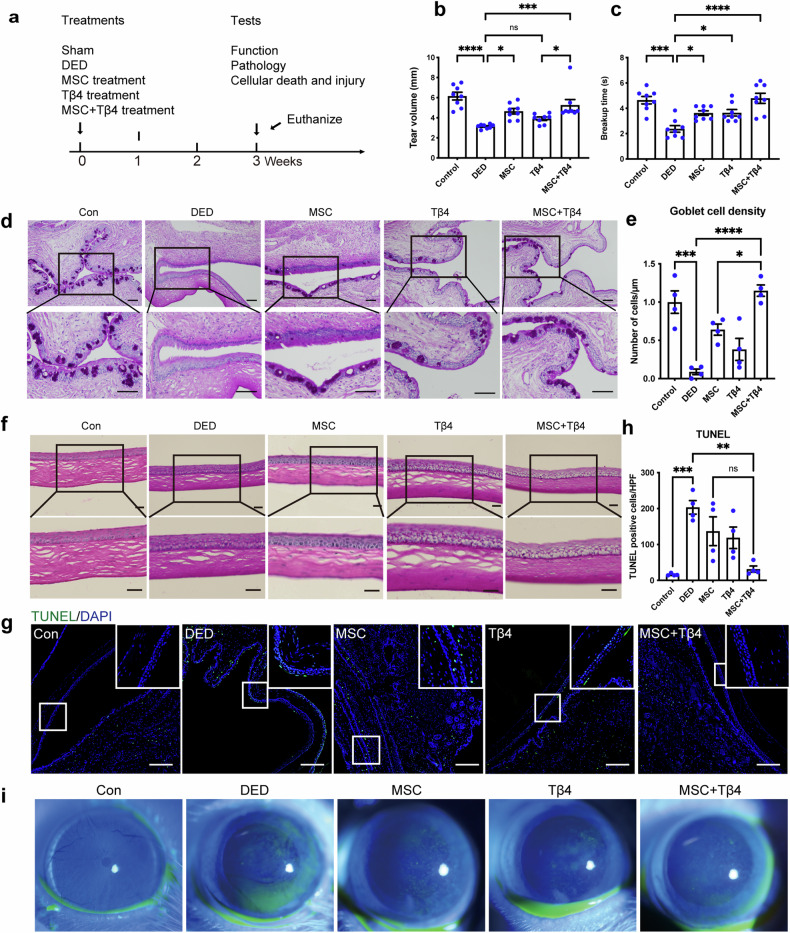
The combination therapy of MSC and Tβ4 exhibited an enhancement in dry eye conditions, contributing to the improved restoration of tear secretion, mitigation of pathological damage, and attenuation of apoptosis. **a** Experimental design. **b** MSC treatment increased tear secretion, and MSC + Tβ4 indicated a substantial enhancement in tear volume, *n* = 8. **c** Both MSC and Tβ4 treatment tended to prolong tear film breakup time (BUT) compared to the DED group, with the combined treatment group more significantly prolonging BUT, suggesting improvement of tear secretion, *n* = 8. The combination treatment increased the number of goblet cells (**d**, **e**) and reduced the pathological damage (**f**), scale bar = 200 μm. **g**, **h** TUNEL staining showed a stronger anti-apoptotic effect following the combination treatment, scale bar = 100 μm. **i** Corneal fluorescein staining images showed a reduction of corneal damage in the combination treatment group. Data are presented as the mean ± SEM. *P < 0.05, **P < 0.01, ***P < 0.001, ****P < 0.0001

To demonstrate the combined therapeutic efficacy of MSCs combined with recombinant Tβ4 in addressing dry eye conditions, we established a rat model of DED through subcutaneous administration of scopolamine hydrobromide into the lower limbs of female Wistar rats^11,29^. These subjects (Fig. 1a) were subsequently categorized into distinct groups: control (Con), DED, MSC treatment (MSC), Tβ4 treatment (Tβ4), and combined MSCs with Tβ4 therapy (MSC + Tβ4). Data indicated that MSC treatment increased tear secretion in model rats, and MSC + Tβ4 indicated a substantial enhancement in tear volume (Fig. 1b). Both MSC or Tβ4 treatment tended to prolong tear film breakup time (BUT) compared to the dry eye group, with the combined treatment group more significantly prolonging BUT (Fig.1c). Concurrently, histopathological assessment of ocular tissue damage was conducted using Periodic acid-Schiff (PAS) staining. The findings showcased a notable reduction in goblet cells within the DED group compared to controls, whereas the combination therapy resulted in an augmented goblet cells and mitigation of both corneal and conjunctival pathology (Fig. 1d-f). This effect was consistently supported by terminal deoxynucleotidyl transferase-mediated dUTP nick-end labeling (TUNEL) staining, which demonstrated heightened anti-apoptotic efficacy in the combination treatment group compared to the monotherapy (Fig. 1g-h). Corneal fluorescein staining images revealed reduced green fluorescence infiltration in the combined therapy, indicative of diminished corneal tissue damage (Fig.1i). Collectively, these findings underscore the potential of the combined Tβ4 and MSC approach against dry eye, yielding enhanced therapeutic efficacy.

### In situ metabolic mass spectrometry indicates glutamine as a key metabolite for combination therapy strategies

**Fig. 2:**
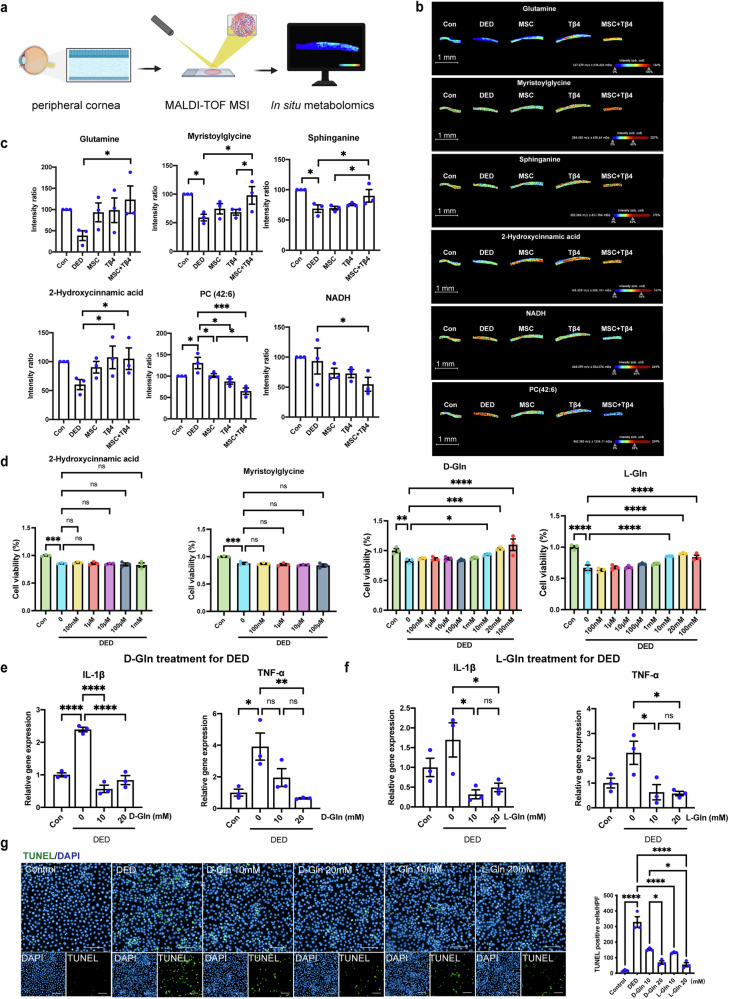
Matrix-assisted laser desorption ionization mass spectrometry imaging (MALDI-MSI) visualized alterations of metabolites on in-situ cornea with/without treatment. **a** The schematic diagram illustrates the in situ metabolomics. The scheme was created with Biorender.com with license. **b** MALDI-MSI visualization of in situ metabolic alterations in the DED group, healthy controls, and the three aforementioned treatment groups. **c** Intensity of metabolites in (**a**). **d** Cell viability via CCK8 showing both D-Gln and L-Gln attenuated corneal epithelial cell damage after DED, while other metabolite treatments did not exhibit enhanced efficacy against corneal cell damage. **e**, **f** Glutamine-treated groups of both conformations (D-Gln and L-Gln) reduced IL-1β and TNF-α confirmed by RT-qPCR. **g** TUNEL staining showed a stronger anti-apoptotic effect in glutamine-treated groups of both conformations (D-Gln and L-Gln) in vitro, scale bar = 50 μm. Data are presented as the mean ± SEM, *n* = 3. *P < 0.05, **P < 0.01, ***P < 0.001

DED is characterized by an adaptive immune-mediated inflammatory response, wherein metabolic pathways are recognized as significant modulators of immune inflammation^4^. To decipher the therapeutic mechanism underlying MSC combined with Tβ4 treatment, we conducted an analysis of in situ metabolic alterations in the DED group, healthy controls, and the three aforementioned treatment groups utilizing MALDI-MSI. This technique facilitates simultaneous assessment of metabolite abundance and its spatial distribution, allowing for precise evaluation of metabolic changes within complex ocular tissue structures. Notably, the peripheral cornea serves as a critical site of dry eye injury, exhibiting a distinct profile from the avascular structure of the central cornea. The peripheral cornea is characterized by a corneal limbal structure, rich in blood vessels and housing a reservoir of pluripotent stem cells. This region represents a transitional zone between the cornea and the sclera or conjunctiva. Employing MALDI-MSI, we investigated in situ metabolic modifications within the peripheral cornea across each treatment group, contrasting with the dry eye group (Fig. 2a). Our aim was to identify key metabolites contributing to the amelioration of dry eye symptoms subsequent to combined treatment.

We have identified a total of 224 metabolites via MALDI/MSI. A total of six distinct metabolites exhibiting variations among the five experimental groups were identified (Fig. 2b, c, Supplementary Figs. 1–2, Supplementary Table. 1). These six metabolites, which stem from the statistically significant differences observed between the DED group and the combination treatment group, suggested that these metabolites may play a potentially critical role in the treatment of dry eye disease. These altered metabolites were linked to pathways of amino acid, sphingolipid, calcium signaling and glycerophospholipids metabolism (Table 1). In our focused analysis comparing metabolic differences between the combination therapy group (MSC + Tβ4) and the DED group, we observed that the combination therapy group displayed elevated levels of four metabolites: glutamine, 2-Hydroxycinnamic acid, myristoylglycine and sphinganine when contrasted with the dry eye group. Conversely, NADH and PC (42:6) exhibited decreased levels relative to the DED group.

**Table 1:** Metabolite identity putatively assigned in rat eye tissues by positive ion MALDI-TOF/TOF MS and LC/MS-MS

Metabolite	Superpathway	Subpathway	DED vs. Con	MSCs+Tβ4 vs. DED
Glutamine	Other of amino acid	Amino acid	Not sig	Increased
2-Hydroxycinnamic acid	-	-	Not sig	Increased
Myristoylglycine	-	-	Decreased	Increased
Sphinganine	Lipid	Sphingolipid	Decreased	Increased
NADH	Signal transduction	Calcium signaling	Not sig	Decreased
PC(42:6)	Lipid	Glycerophospholipid	Increased	Decreased

Metabolites displaying elevated levels may be key participating metabolites in the combination therapy group. To ascertain the specific pivotal metabolites, we applied up-regulated metabolites to an in vitro model of DED to test their effects via cell viability assays, which objective was to observe whether there was any mitigation of dry eye injury (Fig. 2d). Given that glutamine (Gln) exists in two conformations (D-Gln and L-Gln), both conformations of them were tested in DED, and data showed that both D-Gln and L-Gln attenuated corneal epithelial cell damage after dry eye, whereas the other available metabolites failed to revitalize corneal epithelial cells after dry eye injury, suggesting that glutamine as a key metabolite for combination therapy strategies. Furthermore, the RT-qPCR indicated D-Gln and L-Gln ameliorated secretion of inflammatory cytokines in vitro, respectively (Fig. 2e, f). In addition to the inflammatory response, apoptosis is another characteristic injury in DED, thus TUNEL staining was conducted, and Gln application demonstrated a reduction in cell apoptosis following dry eye injury (Fig. 2g). Collectively, this observation implies that glutamine may be a key metabolite in the combined therapeutic strategy of MSCs and Tβ4 against DED.

### Inhibition of glutamine attenuates the protective effects of combined therapy on inflammation, apoptosis inhibition, and homeostasis maintenance

**Fig. 3:**
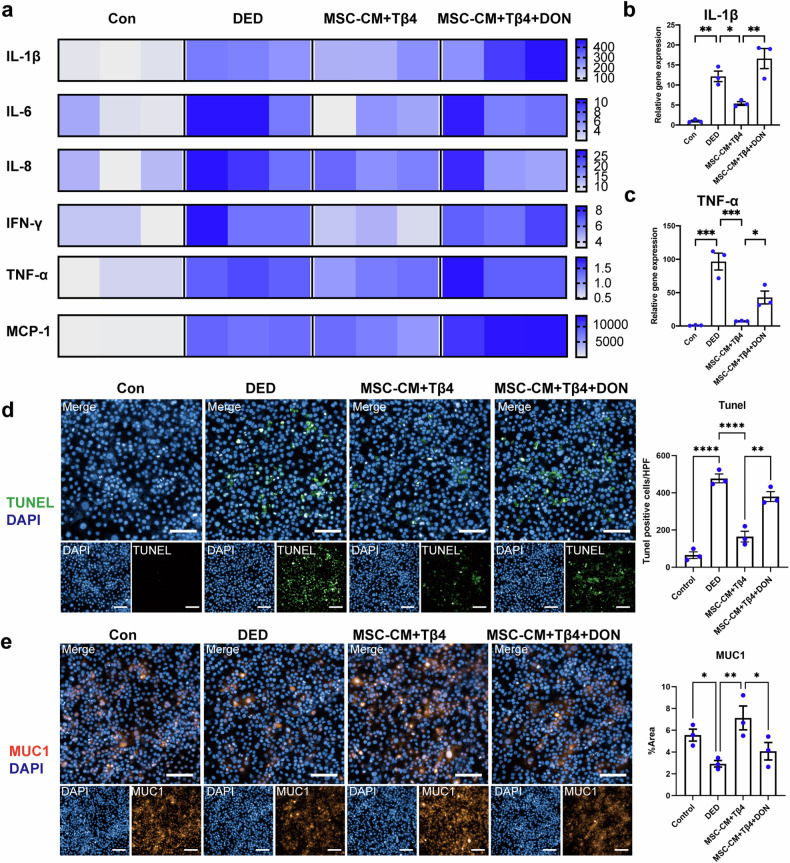
Inhibition of glutamine attenuated the protective effects of combined therapy on inflammation, apoptosis inhibition, and homeostasis maintenance. **a**–**c** High-throughput analysis and RT-qPCR showed an attenuation of inflammatory response by MSC-CM + Tβ4 combination therapy, while the reversal of the beneficial effects was observed upon the glutamine inhibitor DON. **d** TUNEL staining showed the anti-apoptotic effect following the treatment of MSC-CM + Tβ4, whereas DON negated the contributory role of glutamine. Scale bar = 50 μm. **e** Immunofluorescence staining indicated MSC-CM + Tβ4 combination reinstated MUC1 expression in corneal epithelial cells, which is the pivotal factor in maintaining ocular surface homeostasis. Scale bar = 50 μm. Data are presented as the mean ± SEM, *n* = 3. *P < 0.05, **P < 0.01, ***P < 0.001

To further validate glutamine’s pivotal role in the enhanced efficacy of MSC + Tβ4 combination therapy, we employed DON, a special glutamine inhibitor, for intervention in vitro. DON (6-Diazo-5-oxo-L-norleucine, commonly referred to as Diazooxonorleucine or L-6-Diazo-5-oxonorleucine) is an antibiotic isolated from Streptomyces species. It acts as a structural analog of glutamine, functioning as a glutamine antagonist. This compound effectively inhibits the activity of enzymes that utilize glutamine as a substrate by competitively and covalently binding to the active site of these enzymes^30^. Consequently, DON serves as a broad-spectrum inhibitor, inactivating various glutamine metabolic enzymes. This mechanism highlights that DON comprehensively blocks the actions of glutamine through competitive binding at the active site^31^. We employed MSC supernatant (MSC-CM) to replicate the effects of MSCs in an in vitro dry eye model subjected to a hyperosmotic environment. Through high-throughput analysis of inflammatory cytokines, we observed that the combination of MSC-CM and Tβ4 (MSC-CM + Tβ4) curbed the secretion of numerous inflammatory and chemokine factors within the DED group (Fig. 3a). Notably, this dampened inflammatory response was reverted with the application of DON. Alongside protein level assessments, our focus extended to alterations in inflammatory factors at the transcriptional level. The RT-qPCR experiments demonstrated that the MSC-CM + Tβ4 treated group attenuated the expression of IL1β and TNF-α in the context of dry eye. Likewise, the administration of DON reversed these favorable effects (Fig. 3b, c).

Alongside the inflammatory response, apoptosis is a prominent pathological feature in DED. TUENL staining revealed that the anti-apoptotic effect of MSC-CM + Tβ4 on dry eyes was reversed by DON, indicating that the combination treatment improved apoptosis via modulation of glutamine (Fig. 3d).

Further supporting these findings, we assessed the expression of MUC1, a pivotal factor in maintaining ocular surface homeostasis and regulating inflammation^32^. MUC1 (Mucin 1) is identified as a highly glycosylated glycoprotein primarily expressed in epithelial cells, belonging to the mucin family. It constitutes a major component of mucus in ocular surface cells, such as corneal and conjunctival epithelial cells. MUC1 plays a crucial role in maintaining ocular surface health by forming a protective barrier that lubricates the surface, prevents the invasion of pathogens, allergens, and external irritants^33^. Consequently, MUC1 is often utilized as a phenotypic indicator of dry eye disease, reflecting the extent of ocular surface damage^34^. MUC1 is primarily localized in the cornea, conjunctival epithelium, and tears^35^. Dysregulation of MUC1 can result in the absence and denaturation of the glycocalyx mucin barrier, leading to ocular surface alterations and disruption of the dry eye tear film^36^. This, in turn, can contribute to the onset and progression of DED. Several studies have demonstrated that DED patients exhibit reduced MUC1 expression in tears and corneal epithelium, with a positive correlation to disease severity^37^. Immunofluorescence staining for MUC1 (Fig. 3e) confirmed the downregulation of MUC1 expression in the DED group, consistent with previous reports. However, combination therapy significantly restored MUC1 expression in corneal epithelial cells. Notably, the glutamine inhibitor DON reversed this recovery in MUC1 levels. These findings collectively indicate that inhibition of glutamine reversed the protective effects of MSC + Tβ4 combined therapy, suggesting that the MSC + Tβ4 treatment group exerts anti-inflammatory, anti-apoptotic, and homeostasis-maintaining effects on dry eye through glutamine modulation.

### Patient and rat samples suggests the potential involvement of glutaminase 1 (GLS1), an enzyme upstream of glutamine metabolism, in dry eye therapy

**Fig. 4:**
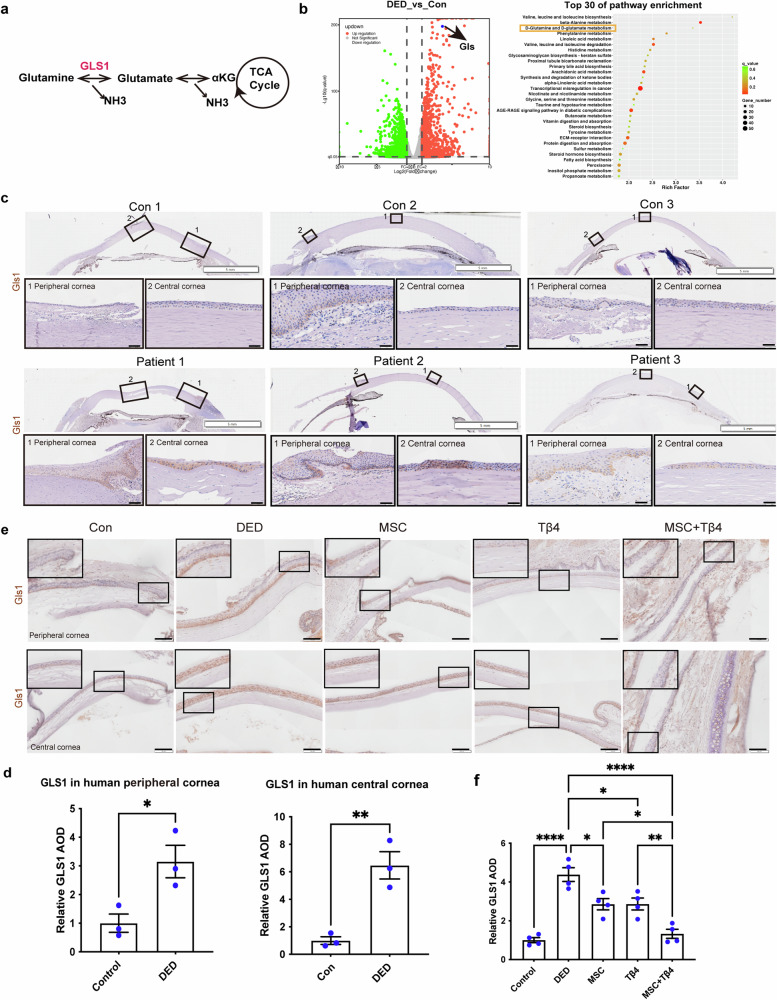
Patient and rat samples suggested the potential involvement of glutaminase 1 (GLS1), an enzyme upstream of glutamine metabolism, in dry eye therapy. **a** Schematic illustration showing that GLS1 is a key enzyme to catalyze glutamine catabolism. **b** RNA-seq analysis of rat DED model suggested that D-glutamine and D-glutamate metabolism were enhanced in the dry eye group and gene GLS is up-regulated. **c**, **d** Immunohistochemical staining of clinical dry eye patients corroborated the significant increase of GLS1 in the cornea, *n* = 3. Scale bar = 50 μm. **e**, **f** A rat model of dry eye verified that GLS1 was up-regulated in the dry eye group, whereas the MSC + Tβ4 combination treatment reduced GLS1 expression, *n* = 3. Scale bar = 100 μm

In our previous discussion, we observed that the MSC + Tβ4 combination treatment mitigated DED injury via upregulating glutamine. However, the mechanism through which combination therapy restored glutamine levels remained unclear. To address this, we revisited the regulating glutamine metabolic pathways and identified glutaminase 1 (GLS1), an enzyme responsible for catalyzing the catabolism of glutamine to glutamate, may serve as a central player (Fig. 4a). Notably, our transcriptomic data revealed (Figs. 4b, S3) that the metabolic pathway “D-glutamine and D-glutamate” in corneal epithelial cells was significantly enriched in dry eye group compared to the control. Moreover, within this pathway, the gene GLS1 exhibited elevated expression in the dry eye group. We further validated these findings using clinical samples, and immunohistochemical staining of the clinical specimens confirmed the upregulation of GLS1 expression in the corneas of dry eye patients when compared with controls (Fig. 4c, d, Table S2). These results suggested that GLS1 may represent a potential key target in DED.

Similarly, the immunohistochemical staining of rats (Fig. 4e, f) demonstrated a significant increase in GLS1 expression in the DED group compared to the normal one, aligning with our transcriptome data. To elucidate whether the therapeutic effects of the combination therapy were mediated by GLS1 modulation, we evaluated GLS1 expression in each treatment group. Notably, the expression of GLS1 was reduced in both the MSC and Tβ4 treatment groups alone compared to the DED group. Interestingly, the MSC + Tβ4 combination therapy further downregulated GLS1 expression, bringing it nearly in line with the control group. These observations suggest GLS1 may be a key target for the combination treatment to ameliorate dry eye.

### The combination therapy alleviates dry eye damage by inhibiting GLS1 to restore glutamine levels, inhibiting downstream inflammation in DED

**Fig. 5:**
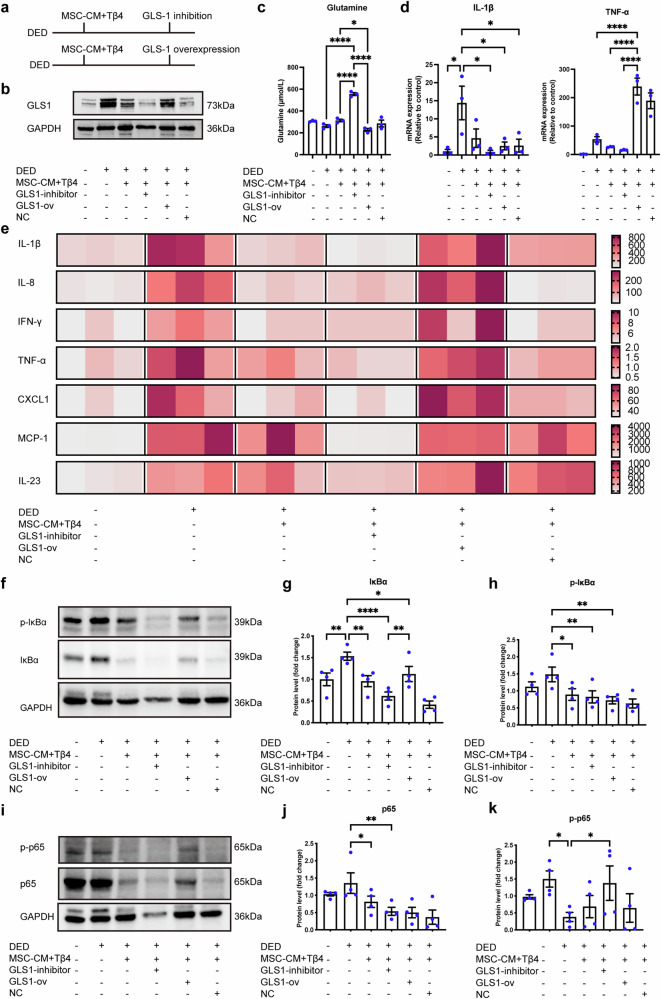
The combination therapy alleviated dry eye damage by inhibiting GLS1 to restore glutamine levels, inhibiting downstream inflammation in DED. **a** Experimental design of inhibition and overexpression of GLS1. **b** Western blot confirmed the inhibition and overexpression of GLS1. **c** Inhibition of GLS1 restored the glutamine levels. **d**, **e** The RT-qPCR and high-throughput analysis showed an attenuation of inflammatory response by MSC-CM + Tβ4 combination therapy, while overexpression and inhibition of GLS1 reversed/augmented the effects of MSC + Tβ4 on inflammation, respectively. Data are presented as the mean ± SEM, *n* = 3. **f**–**k** Western blotting showed that MSC + Tβ4 down-regulated the IκBα/NF-κB pathway by inhibiting GLS1. Data are presented as the mean ± SEM, *n* = 4. *P < 0.05, **P < 0.01, ***P < 0.001, ****P < 0.0001

In order to further investigate whether MSC + Tβ4 combination therapy can alleviate DED damage by inhibiting GLS1, interventions targeting GLS1 overexpression and inhibition were conducted in an in vitro dry eye model (Fig. 5a). The successful execution of overexpression and inhibition interventions on GLS1 was depicted in Fig. 5b. Further data showed that inhibition of GLS1 restored the glutamine levels (Fig. 5c). To explore the downstream inflammatory responses, the RT-qPCR was conducted and demonstrated that MSC + Tβ4 reduced the expression of inflammatory factors in the DED group, however, when GLS1 was overexpressed alongside combination treatment, this anti-inflammatory effect was reversed (Fig. 5d). Conversely, inhibiting GLS1 during combination therapy showed a numerical decrease in inflammatory factor expression, although not statistically significant compared to the treated group. Additionally, we examined the protein expression levels of these inflammatory factors. High-throughput analysis of various inflammatory factors and chemokines (Fig. 5e) revealed that MSC-CM + Tβ4 reduced their expression in the DED group. However, when GLS1 was inhibited or overexpressed, the levels of these factors in corneal epithelial cells were reduced and increased, respectively. In other words, inhibition and overexpression of GLS1 enhanced and reversed the anti-inflammatory effects of combination treatment.

Similarly, the results from immunoblotting experiments (Fig. 5f–k) indicated that both total and phosphorylated forms of IκBα and NF-κB were downregulated in MSC-CM + Tβ4 compared to the DED group. However, inhibiting and overexpressing GLS1 respectively strengthened and reversed the inhibitory effects of the combination treatment on the IκBα/NF-κB pathway. Consequently, the combination therapy modulated GLS1 to restore glutamine levels, inhibiting downstream IκBα/NF-κB signaling and the expression of associated inflammatory factors, thereby alleviating dry eye injury.

### Single-cell dissection of cellular and molecular features underlying glutamine therapy in dry eye disease

**Fig. 6:**
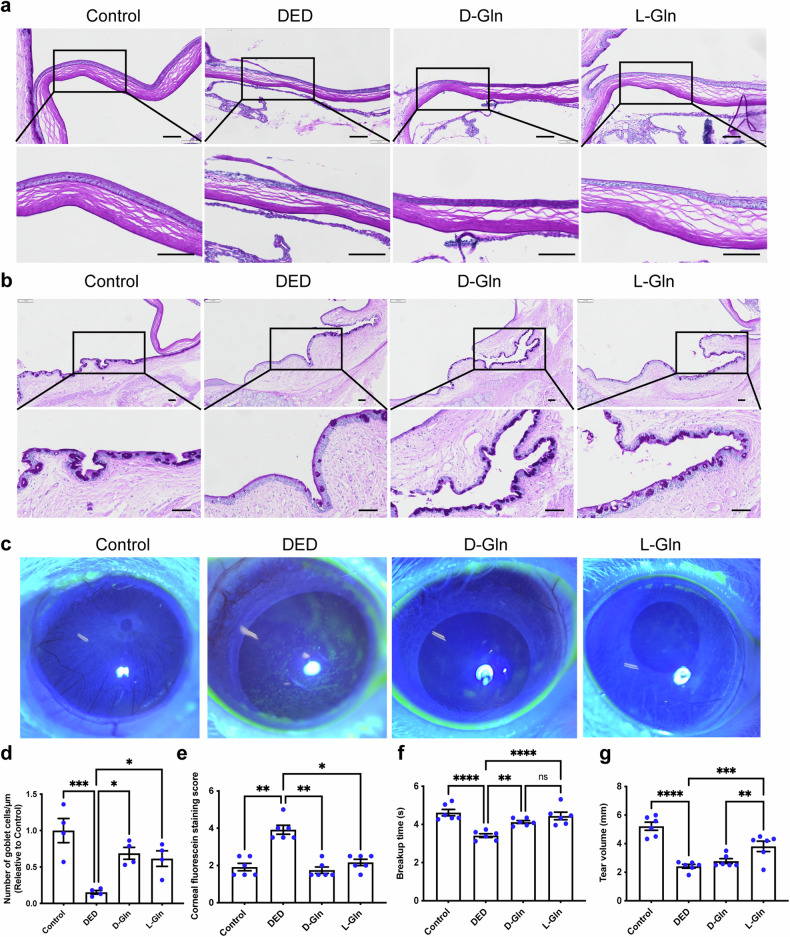
The therapeutic efficacy of glutamine against DED in vivo. **a** The in vivo efficacy of glutamine in dry eye was initially validated. Rats were randomly divided into control group (Control), dry eye disease (DED) model group (PBS as solvent), D-Gln, and L-Gln treatment groups. PAS staining showed that Glutamine therapy reduced the pathological damage. Scale bar = 100 μm. **b** Glutamine therapy increased the number of goblet cells. Scale bar = 100 μm. **c** Corneal fluorescein staining images showed a reduction of corneal damage in glutamine-treated group. **d** Quantification of the expression of goblet cells in (**b**). **e** Quantification of the corneal fluorescein staining in (**c**). Functional testing of tear secretion revealed both D-Gln and L-Gln treatment groups prolonged tear film breakup time (**f**), with the L-Gln treatment demonstrating superior restoration of tear volume (**g**). Data are presented as the mean ± SEM. *P < 0.05, **P < 0.01, ***P < 0.001, ****P < 0.0001

To explore the cellular and molecular program driving the progression of dry eye disease and how glutamine affect the inflammation of DED, we tended to perform unbiased single-cell RNA sequencing (scRNA-seq) on control eyes, DED models and glutamine-treated group. Before conducting scRNA-seq, the in vivo efficacy of glutamine in dry eye was initially validated. Rats were randomly divided into control group (Control), DED model group (PBS as solvent), D-Gln, and L-Gln treatment groups. Pathological evaluation of ocular tissue damage was performed using PAS staining, revealing that both D-Gln and L-Gln configurations mitigated corneal damage (Fig. 6a) and increased conjunctival goblet cell counts (Fig. 6b, d). Corneal fluorescein staining images and scores indicated reduced fluorescence infiltration post-treatment with both glutamine configurations, suggesting alleviation of corneal tissue damage (Fig. 6c, e). Functional testing of tear secretion revealed that both D-Gln and L-Gln treatment groups prolonged tear film breakup time (BUT), with the L-Gln treatment group demonstrating superior restoration of tear volume (Fig. 6f, g).

**Fig. 7:**
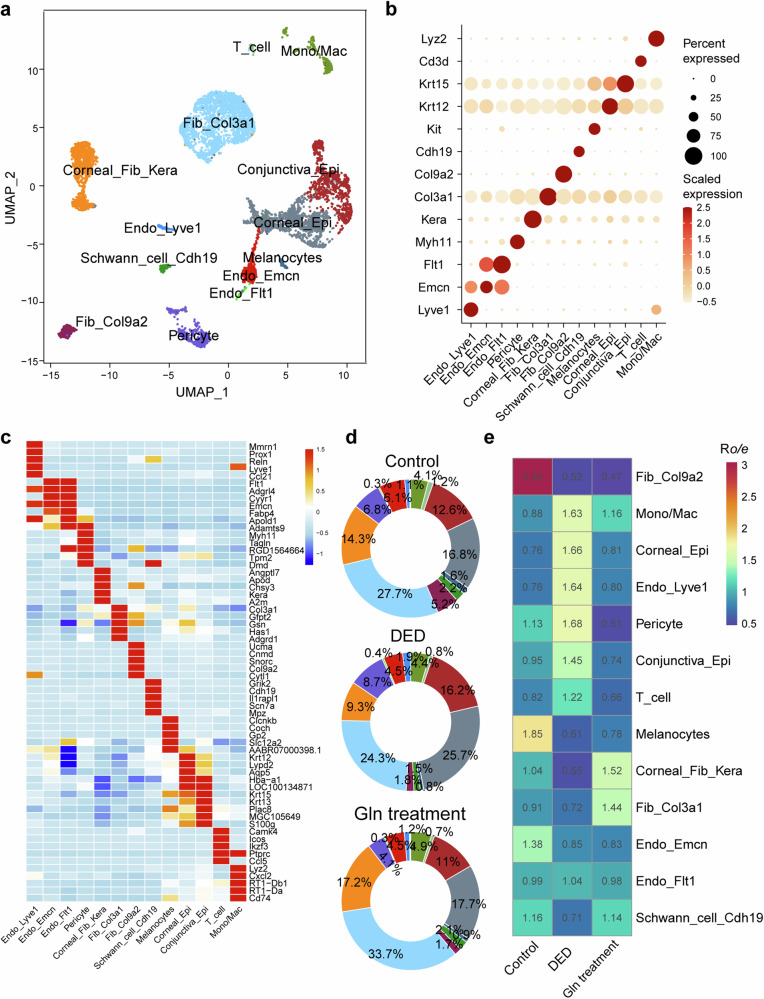
Single-cell sequencing revealed dynamic cellular characteristic and changes in the proportionality of cell types in rat DED model with glutamine treatment. Distribution of the major cell types (**a**) based on the expression of top 5 differentially expressed genes (**b**) and cell-type specific markers (**c**). **d**, **e** The relative prevalence of each cell types across the control, DED and Gln treatment group. The DED model group revealed remarkable enrichment of immune cells such as T cells, B cells, and corneal epithelial cells (Corneal_Epi) while the Gln therapy reduced the immune cell infiltration and the percentage of Corneal_Epi

Following confirmation of glutamine efficacy in the aforementioned in vivo experiments, unbiased scRNA-seq was performed on 3 normal control rat eye samples, 3 DED model rat eye samples, and 3 DED model rat eye samples receiving glutamine therapy. In total, 70,100 cells were adopted for further bioinformatics analysis after religious quality control, which can be decomposed into 13 major cell types (Fig. 7a) based on the expression of top 5 differentially expressed genes (DEGs, Fig. 7b, Table S3) and cell-type specific markers (Fig. 7c), comprising Lyve1^+^ endothelium (marked by Lyve1, Endo_Lyve1), Emcn^+^ endothelium (marked by Emcn, Endo_Emcn), Flt1^+^ endothelium (marked by Flt1, Endo_Flt1), Corneal fibroblast (marked by Kera, Corneal_Fib_Kera), Pericyte (marked by Rgs5), Col3a1^+^ fibroblast (marked by Col3a1, Fib_Col3a1), Col9a2^+^ fibroblast (marked by Col9a2, Fib_Col9a2), Schwann cell (marked by Cdh19), Melanocytes (marked by Kit), Corneal epithelium (marked by Krt12, Corneal_Epi), Conjunctiva_Epi (marked by Krt13/14/15, Conjunctiva_Epi), T_cell (marked by Cd3d), and Mono/Mac (marked by Lyz2). We further compared the relative prevalence of each cell types across three groups and found that the DED model group revealed remarkable enrichment of immune cells such as T cells, B cells, and Corneal_Epi, while the DED model receiving glutamine therapy could reduce the immune cell infiltration and the percentage of Corneal_Epi (Fig. 7d, e).

**Fig. 8:**
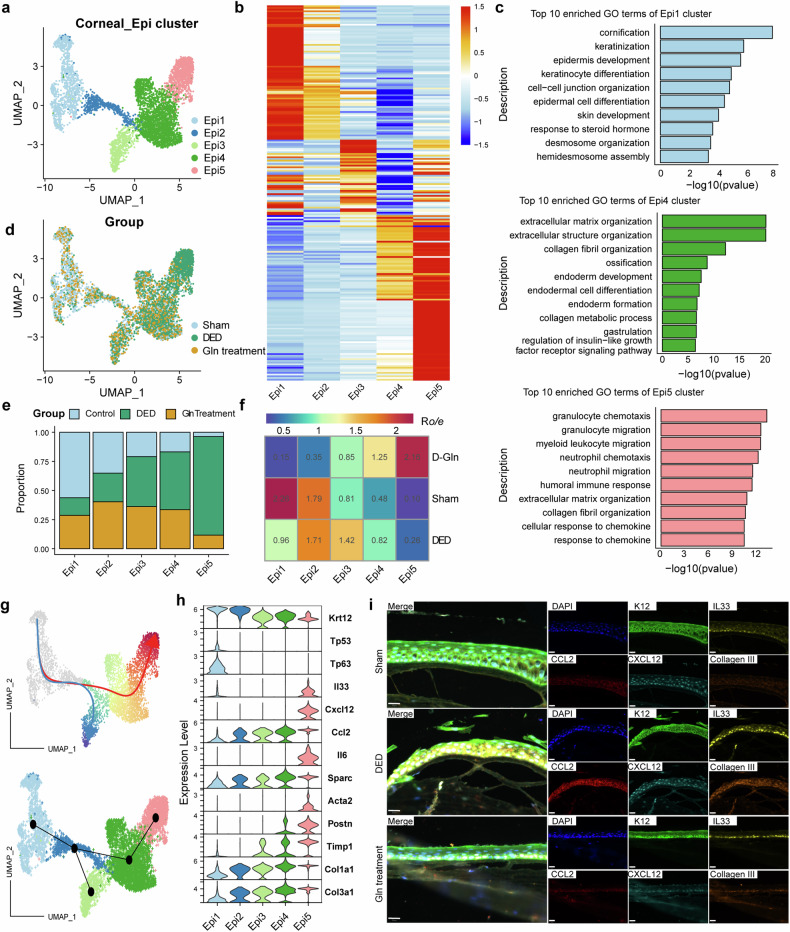
Glutamine treatment reduced the proportion of pro-inflammatory and pro-fibrotic corneal epithelium, reducing their inflammatory cytokine secretion. Sub-clustering analysis of total corneal epithelial cells generated 5 subclusters (**a**) with different expression pattern (**b**). **c** The GO term enrichment analysis on the top 50 DEGs of corneal Epi1, Epi4, and Epi5. **d** The distribution of each corneal epithelium subcluster across three sample groups. **e** Barplot showing the percentage of each corneal epithelium subcluster across three sample groups. **f** The odds ratios (Ro/e) revealed the cell distribution of corneal epithelium subcluster using the STARTRAC-dist index method. **g** The differentiation routes of corneal epithelial cells revealed by slingshot trajectory analysis. The corneal epithelial cells exhibited two differentiation trajectories, routine one was from Epi1 to Epi3, and the other one (routine two) was from Epi1 to Epi3 and then to Epi4, and Epi5. **h** The marker gene expression of 5 subclusters generating from corneal epithelial cells. **i** Typicle multiplexed immunofluorescence histological staining of corneal epithelial cells from the control, DED and Gln-treated group. Data indicated Gln treatment achieved reduction of inflammatory factors IL33, CXCL12 and CCL2 compared to the DED group, along with decreased fibrosis marker Collagen III expression

As the Corneal_Epi the most important cell type, which was influenced by the inflammation, we next mainly focused on the molecular diversity of Corneal_Epi, and further sub-clustering analysis of total Corneal epithelial cells generated 5 subclusters (Fig. 8a) with different expression pattern (Fig. 8b, Table S4). The GO term enrichment analysis on the top 50 DEGs of each Corneal_Epi subcluster revealed that Epi1 was enriched for keratinocyte differentiation, epidermal cell differentiation, epidermis development, which was associated with corneal epithelium development. While the Epi4 and Epi5 was enriched for extracellular matrix organization, collagen fibril organization, which indicated a profibrotic phenotype, and the Epi5 was also enriched for the inflammation associated GO terms such as myeloid leukocyte migration, cellular response to chemokine, response to chemokine, indicated that Epi5 was proinflammatory and pro-fibrotic (Fig. 8c). We also compared the relative prevalence of each Corneal epithelium subcluster across three groups and found that the DED model group revealed remarkable enrichment of proinflammatory and pro-fibrotic Epi4 and Epi5, while the DED model receiving glutamine therapy could reduce the percentage of Epi4 and Epi5 (Fig. 8d–f). Interestingly, we found that the corneal epithelial cells exhibited two differentiation trajectories, routine one was from Epi1 to Epi3, and the other one (routine two) was from Epi1 to Epi3 and then to Epi4, and Epi5 (Fig. 8g, h). The expression of classic corneal epithelial cells marker Krt12 decreased dramatically alongside the routine two differentiation trajectory, while the proinflammatory cytokines and chemokines such as Il33, Cxcl12, Ccl2, Il6, and the pro-fibrotic signatures Sparc, Timp1, Col3a1, Col1a1 showed opposite expression trend, and were both highly upregulated alongside the routine two differentiation trajectory.

To validate these results from scRNA-seq, multiplexed immunofluorescence histological staining was performed (Fig. 8i). The K12 staining was used to localize corneal epithelial cells, observations revealed that in the DED group, epithelial cells exhibited upregulation of inflammatory factors IL33, chemokines CXCL12 and CCL2 compared to the Sham group, along with increased fibrosis marker Col3a expression. Following treatment with Gln, the expression of these inflammatory and fibrotic markers in epithelial cells decreased, validating our single-cell sequencing findings. Therefore, the above results indicated that the corneal epithelial cells achieved the proinflammatory and pro-fibrotic phenotype during the DED progression, while the glutamine could relieve symptoms by inhibiting the inflammation and fibrosis of corneal epithelial cells.

## Discussion

In this study, we proposed and assessed the efficacy of combining MSCs and Tβ4 therapies in restoring glutamine levels. This restoration was achieved by inhibiting glutaminase, followed by further inhibition of the downstream IκBα/NF-κB pathway and subsequent reduction in inflammatory factor expression. As a result, dry eye disease was ameliorated. Our findings collectively suggested a previously unrecognized, pivotal role of GLS1 and glutamine metabolism in the realm of DED. Moreover, these insights offered both theoretical foundations and an appealing strategy for DED treatment (Fig. 7).

Dry eye, a prevalent ocular surface disorder, is typically addressed with primary therapies like artificial tears and anti-inflammatories (corticosteroids, cyclosporine, lifitegrast)^1^. However, these current treatments remain palliative^38,39^, while certain conservative approaches show limited efficacy in some cases^38^. Additionally, drug-related side effects such as ocular burning and high costs impede the clinical application of current therapies^3,38^. Consequently, the imperative to develop novel alternative therapies explicitly targeting dry eye disease continues to gain momentum. MSCs, known for their potential in restoring tissue function and displaying robust anti-inflammatory efficacy, have found utility across diverse regenerative strategies^40,41^. Prior research has presented multiple reports on the efficacy of MSCs in treating DED. MSC transplantation has exhibited promising results in ameliorating dry eye inflammation, including improved lacrimal gland regeneration in a mouse model of DED, accompanied by the suppression of macrophage infiltration and the expression of inflammatory factors^42–44^. The efficacy of MSCs in addressing aqueous tear deficiency (ATD) was initially demonstrated in a canine model^45^. Moreover, an open-label study administering a transconjunctival injection of adipose-derived stem cells to seven ATD subjects showed significant enhancements in clinical parameters from baseline^46^. Our study advances prior stem cell-based DED interventions by integrating MSC treatment with our co-developed recombinant human Tβ4^47^.

Tβ4 can exert an immunomodulatory effect, which reduces the release of cytokines such as IL-1 receptor-associated kinase by regulating the NF-κB and Toll-like receptor pathways, thereby attenuating inflammatory injury^48^, it also has been reported to have a certain mitigating effect on inflammation in dry eyes as well^26^. In addition, Tβ4 promotes the proliferation and enhances the repair ability of stem cells^28^. Therefore, the combination therapy of MSC with Tβ4 combination therapy can better enhance the immunomodulatory effect of MSC, alleviate corneal epithelial damage caused by DED, and improve key clinical indicators such as tear volume and BUT in DED, providing a new strategy and theoretical basis for stem cell therapy.

As to how MSCs and Tβ4 specifically modulate IκBα/NF-κB pathway, previous studies have demonstrated that MSCs exert significant paracrine effects that inhibit the binding of lipopolysaccharides to the Toll-like receptor 4 (TLR4) complex, particularly via the TLR4/CD14 pathway. This modulation subsequently influences IκBα and AP-1 transcriptional activity, leading to a reduction in the release of inflammatory cytokines^49^. Furthermore, MSCs have been shown to effectively target tumor necrosis factor-stimulated gene 6 (TSG-6), which inhibits the NF-κB/NLRP3 signaling pathway and regulates the phenotypic transformation of macrophages^50^. Notably, MSCs can suppress p65 phosphorylation, NF-κB transcriptional activity, and histone deacetylase 3 (HDAC3) expression, thereby alleviating neuroinflammation^51^. In addition, MSCs inhibit the proliferation and cytotoxicity of natural killer (NK) cells, as well as the differentiation and maturation of dendritic cells (DCs). They also modulate neutrophil infiltration and apoptosis through the secretion of various cytokines, including IL-10, HLA-G, TGF-β, prostaglandin E2, indoleamine 2,3-dioxygenase (IDO), and inducible nitric oxide synthase (iNOS)^52^. As for Tβ4, In a corneal epithelial debridement model involving rats, Tβ4 was observed to significantly reduce pro-inflammatory mediators and chemokines. Specifically, Tβ4 effectively diminished TNF-α-induced NF-κB activation in corneal epithelial cells. Compared to untreated controls, Tβ4 treatment notably lowered nuclear NF-κB protein levels, NF-κB activity, and the phosphorylation of the p65 subunit in TNF-α-stimulated corneal epithelial cells, thus underscoring the potential clinical applications of Tβ4 as an anti-inflammatory agent^53^.

The therapeutic effectiveness of MSCs and Tβ4 may be significantly affected by both the ocular microenvironment and systemic factors^54,55^. Notably, the microenvironment of the ocular surface differs fundamentally from that of other anatomical locations, characterized by increased permeability and distinct immune properties. This ocular microenvironment encompasses not only the components of tears but also various cytokines and growth factors, as well as the intricate interactions among corneal epithelial, stromal, and endothelial cells^56^. These unique features can directly influence the migration, proliferation, and differentiation capabilities of MSCs, thereby impacting their therapeutic outcomes. Specifically, the functionality of MSCs within the ocular microenvironment may be modulated by factors such as the severity of corneal injury, the inflammatory state, and interactions with other cell types^57^. Consequently, the condition of the ocular surface microenvironment plays a critical role in regulating the anti-inflammatory effects and regenerative potential of MSCs. Moreover, systemic factors must be considered. Patients with dry eye syndrome often present with comorbid systemic conditions, such as diabetes and autoimmune disorders, which can elicit a systemic inflammatory response that adversely affects the ocular microenvironment and local treatment efficacy. For example, hyperglycemic conditions may impair corneal regeneration and potentially diminish the effectiveness of MSCs and Tβ4. Furthermore, inter-individual variations, including genetic predispositions, age, and sex, may contribute to differences in the ocular microenvironment and the resulting treatment responses among patients.

This study also looks into the mechanism of MSC + Tβ4 from a metabolic perspective via MALDI-MSI. In contrast to conventional metabolomics, which merely offers semiquantitative data regarding metabolite abundance, devoid of spatial distribution insight, MALDI-MSI enables label-free quantification of metabolites directly within tissue sections. The intricate variations in ocular tissue substructures, coupled with diverse metabolic profiles across tissue regions, could potentially hinder comprehensive comprehension of dry eye pathogenesis unless quantitative and localized metabolite analyses are concurrently conducted. Our prior utilization of MALDI-MSI for in situ analysis efficiently unveiled region-specific metabolic dynamics in DED, thereby shedding light on DED pathogenesis^11^. In this present investigation, our focus on the peripheral cornea, a central site of dry eye injury, facilitated the in-situ visualization of metabolic shifts pre- and post-combined treatment via MALDI-MSI. This showed the pivotal role of glutamine and its upstream key enzyme GLS1 in dry eye treatment, elucidated the molecular foundation of MSCs and Tβ4 combination therapy, and unveiled potential novel targets for dry eye treatment.

This study presents the novel finding that glutamine can modulate inflammation associated with DED. To validate this assertion, we employed single-cell RNA sequencing to perform a comparative analysis of corneal epithelial cells from DED patients and healthy controls. Our results identified five distinct subpopulations of cells exhibiting different expression patterns. Notably, during the progression of DED, particularly the fourth (Epi4) and fifth (Epi5) subpopulations were significantly enriched, while treatment with glutamine effectively reduced the proportion of these cell subpopulations. Subsequent differential gene expression (DEG) and Gene Ontology (GO) enrichment analysis revealed that the Epi4 and Epi5 subpopulations were enriched for terms related to extracellular matrix (ECM) and collagen fiber. This finding suggests that Epi5 possesses characteristics associated with fibrosis. Furthermore, Epi5 was also enriched in various inflammation-related GO terms, including myeloid cell migration and cellular responses to chemokines, underscoring its pivotal role in promoting both inflammation and fibrosis. Importantly, the integration of single-cell sequencing data with multiplex fluorescent staining results demonstrated that following glutamine treatment, the expression levels of pro-inflammatory cytokines and chemokines, such as IL-33, CXCL12, and CCL2, as well as fibrosis markers COL3A1 and COL1A1, were significantly downregulated. Additionally, transcriptomic sequencing confirmed the downregulation of the IκBα/NF-κB pathway.

Glutamine, a non-essential amino acid, plays diverse biological effects and participates in a broad spectrum of cellular metabolism and physiological functions^58^. It’s fundamental in trauma, sepsis, and infected states due to its possession of anti-inflammatory, antioxidant, and anti-apoptotic properties^59^. Significantly, glutamine metabolism plays a pivotal role in innate immunity, contributing to T cell activation, regulation of Th1 and Th17 cell differentiation, and integral participation in T cell dynamic homeostasis^60^. The substantial body of evidence underscores glutamine’s protective potential across a range of immunoinflammatory disorders, encompassing psoriasis, cutaneous inflammation, rheumatoid arthritis, and inflammatory bowel disease^59–62^. Consistent with preceding studies, our current investigation corroborates that the combination of MSCs and Tβ4 mitigates DED-induced inflammatory response and corneal epithelial apoptosis by reinstating glutamine expression. Notably, similar efficacy is observed with exclusive glutamine treatment. Furthermore, our findings align with recent research highlighting glutamine’s significance in corneal epithelial injury healing. Specifically, glutamine treatment effectively attenuates endotoxin-induced canine corneal ulceration by suppressing the NF-κB pathway, alongside its downstream TNF-α and IL-6 signaling^63^. Acknowledging the well-established premise that chronic inflammatory responses underlie the core pathophysiological mechanism of DED, our present study, focused on anti-inflammation, establishes the intricate nexus between glutamine metabolism and inflammation. Remarkably, though limited studies have explored glutamine as a direct intervention for corneal injury, our investigation underscores the potential utility of glutamine for dry eye treatment. Notably, unlike other metabolites such as Sphinganine and Cytidine triphosphate (found in Fig. 2) which has little information in biocompatibility and lack accessible commercial sources, thus limiting their feasibility for potential therapeutic applications, glutamine emerges as a promising small molecule therapeutic option, given its inherent endogenous presence, solubility, availability, and favorable biocompatibility and safety profile.

Metabolism-related enzymes play important roles in the regulation of inflammation. Glutaminase plays a key role in glutamine metabolism, as the rate-controlling enzyme catalyzing the hydrolysis of glutamine to glutamate, which further undergoes metabolism to α-ketoglutarate^64^. Mammals exhibit two primary glutaminase variants, namely “renal” glutaminase (GLS1) and “hepatic” glutaminase^65^. In this study we uniquely demonstrate abnormal upregulation of GLS1 expression in both DED patients and rat models, substantiating GLS1-driven glutamine reduction as a vital facet of the dry eye inflammatory response, as supported by GLS1 overexpression and inhibitor experiments. Our findings are consistent with the work of Feng et al., who reported that aberrant GLS1 upregulation in a mouse alkali burn model promoted macrophage infiltration and the secretion of inflammatory mediators, culminating in corneal neovascularization^66^. Notably, some previous studies also showed that mice with GLS1 deficient microglia had attenuated neuoinflammation and decreased the levels of pro-inflammatory cytokines when treated with LPS^67^. Conversely, upregulation of GLS1 has been shown to contribute to impairment neuroinflammation^68^. Furthermore, we elucidated that GLS1 overexpression counteracts the therapeutic benefits of MSC and Tβ4 co-treatment for corneal epithelial injury, whereas BPTES inhibitor application exhibits a reduction in inflammatory factors such as IL-1β and TNFα, consequently ameliorating corneal injury. In line with our observations, Jiang et al. posited that stem cell-derived extracellular vesicles could mitigate osteoarthritis by modulating glutamine metabolism, subsequently curbing GLS1 expression^21^. Collectively, our findings underscore the ability of Tβ4-combined MSCs to impede glutamine degradation via GLS1 modulation, consequently ameliorating inflammatory responses in DED. Moreover, GLS1 inhibitors hold promise as a potential therapeutic avenue for DED.

Above these results suggest that GLS1 plays a key role in DED-mediated inflammation, but the role of GLS1 in corneal inflammation remains unclear. Interestingly, we found that GLS1 regulated inflammation and promoted cytokine production by inhibiting IκBα activation. Through rescue experiments, we demonstrated that GLS1 overexpression increasing IκBα activation and promoting inflammatory cytokine production. Previous studies have shown that genes positively correlated with GLS1 expression were found to be enriched in the NF-κB signaling pathway via transcriptome sequencing data analysis, which is consistent with our results^69^. Moreover, Gao et al. proposed that GLS1, as a mitochondrial enzyme having a key role in cellular bioenergetics and metabolism, may control the generation of reactive oxygen species (ROS) which exacerbating oxidative stress and activating NF-κB signaling pathway^70^. However, dysregulation of glutamine/GLS1 expression could indeed be both a cause and a consequence of the disease, and thus subsequent exploration of whether this dysregulation is causally related to DED is warranted.

This research confirms that a combined treatment approach yields significantly greater therapeutic efficacy than monotherapy. Utilizing Matrix-Assisted Laser Desorption/Ionization Mass Spectrometry Imaging (MALDI-MSI), we directly detected and visualized significant metabolic pathway alterations in situ within the corneal region. This allowed us to further identify the critical role of glutamine metabolism in mediating synergistic effects. Moreover, we established that the exogenous addition of glutamine can achieve therapeutic outcomes comparable to those of the combined treatment. Thus, this study advances our understanding of therapeutic efficacy through a foundational research framework that employs combination therapy. By leveraging cutting-edge technology, we have uncovered and validated new mechanisms and targets, which can be translated into biomarkers and therapeutic eye drops that serve both diagnostic and therapeutic purposes. This innovative approach provides a new perspective and research paradigm for the prevention and treatment of other ocular surface diseases.

## Materials and methods

### Ethical approval

All rat-related procedures were conducted with approval from the Chinese PLA General Hospital Animal Care and Use Committee (2022-X18-135) and Institutional Animal Care and Use Committee of Capital Medical University, Beijing (AEEI-2024-035). The human eye tissue for immunostaining were collected from Chinese PLA General Hospital, with the approval of the Research Ethics Committee, with ethics committee approval number KY2021-027.

### Animals and treatment

Eight-week-old female wild-type Wistar rats were procured from Beijing Vital River Laboratory Animal Technology Co., Ltd. The rats were housed in a specific pathogen-free environment at a constant temperature of 22 ± 4 °C, with controlled lighting conditions (12-h light-dark cycle). All rat-related procedures were conducted with approval from the Chinese PLA General Hospital Animal Care and Use Committee. The rats were sacrificed by an intraperitoneal injection of 100 mg/kg sodium pentobarbital overdose^71^.

### MSC sources, culture, and administration

Human MSCs were isolated from human placentas as previously described under approval of the Ethics Committee of the Chinese Academy of Medical Sciences and Peking Union Medical College and informed consent of each donor, and culture-expanded as previously reported^72–74^. MSCs of passage 5 were used for in our experiments. Cells were cultured in a humidified incubator with 5% CO_2_ at 37 °C and passaged with trypsin/EDTA after reaching the confluence. MSCs were resuspended in HBSS and injected to the conjunctival sac of rats^75^. The therapeutic dosage of MSCs was informed by previously published studies. Specifically, we administered 10^5^ cells per unilateral injection to the conjunctival sac of rats^75^.

### Dry eye disease induction and treatment

The Wistar rats were randomly divided into five groups: the control group, DED group, Tβ4 treatment group, and MSCs treatment group, MSC + Tβ4 treatment group, with 8 animals per group. The induction of DED followed previous protocols, as described in bibliography, involving subcutaneous injections of scopolamine hydrobromide (4.5 mg/mL) four times daily for 2 mL total volume over 21 days^11,29^. The MSCs were injected to the conjunctival sac of rats at a dose of 10^5^ cells per eye every three day. The Tβ4 treatment group was administered 0.1% Tβ4 eye drops thrice daily, in single doses of 25 μL. Additionally, the combined treatment group received both Tβ4 eye drops thrice daily and MSCs (1 × 10^5^ cells per eye, 2 × 10^5^ cells per **rat**, single dose of 25 μL) to the conjunctival sac every three day.

### Tear volume measurement

Tear volume was assessed using a sterile phenol red-soaked cotton thread (ZoneQuickTM) after 21 days of scopolamine hydrobromide injection^76^. The threads were placed in the lower palpebral conjunctiva for 60 s, during which they absorbed tears and turned red. Each eye of the Wistar rats underwent three assessments, and the final length of the red wetted section was recorded as the average, with a ruler. Measurement error was ±0.5 mm.

### Tear Film Breakup Time (BUT) Measurement

BUT was determined as previously outlined^77^. Briefly, 10 μL of liquid sodium fluorescein (Sigma-Aldrich, St. Louis, MO, USA, 10 mg/mL) were instilled into the lower conjunctival fornix. Rats were prompted to blink three times before measurement. Using a slit-lamp equipped with a cobalt blue filter, the corneas of all rats were observed. The time from the last blink to the appearance of the first dark spot on the cornea was recorded. This process was repeated three times for each eye, and the average BUT was recorded.

### Tissue staining

Eye tissues were fixed with 4% paraformaldehyde, followed by paraffin embedding and sectioning at a thickness of 12 μm for periodic acid-Schiff (PAS) and hematoxylin and eosin (HE) staining. Slides were examined using an Olympus microscope. Apoptotic cells were detected through the terminal deoxynucleotidyl transferase-mediated dUTP nick-end labeling (TUNEL) assay using commercial kits (Roche, Basel, Switzerland).

### MALDI-MSI experiment

Fresh-frozen eye samples were sectioned into 12-μm slices using a cryomicrotome at −20 °C (Leica CM1950; Leica Microsystems, Wetzlar, Germany). The sections were placed on a pre-cooled ITO conductive glass slide and vacuum-dried for 20 min. After marking with a Teach Maker, the ITO slides were scanned at a resolution of 7200 dpi. Subsequently, an electric field-assisted circulation matrix sprayer was utilized to uniformly apply a layer of matrix. The matrix employed was 5 mg/mL CHCA (α-cyano-4-hydroxycinnamic acid), dissolved in a 50% methanol aqueous solution containing 0.2% TFA, with a substrate spray volume of 1 mL.

Mass spectrometry imaging was carried out using the rapifleX MALDI-TOF/TOF MS (Bruker Daltonics, Bremen, Germany) equipped with a 10 kHz smartbeam 3D laser^11,78,79^. The settings were as follows: Positive ion mode, laser power at 65%, lens voltage at 11.35 kV, reflector voltage at 20.85 kV, covering a mass-to-charge ratio (m/z) range from 100 to 1200 Da. The spatial resolution for imaging was set to 20 μm for the peripheral cornea tissue, and each spectrum was generated from 100 laser shots. The spectrum data were imported into SCiLS lab 2021c (Bruker Daltonik GmbH) for subsequent analysis, with normalization based on the total ion current.

### Metabolite identification

MS/MS fragmentations, performed on the rapifleX MALDI-TOF/TOF MS in the LIFT mode, were employed for detailed structural confirmation of the identified metabolites. Additionally, LC-MS/MS analyses were conducted using the Thermo Scientific™ Dionex™ MultiMate™ 3000 Rapid Separation LC (RSLC) system coupled to a Q Exactive™ hybrid quadrupole Orbitrap mass spectrometer (Thermo ScientificTM). MS analyses were carried out with a Q Exactive™ hybrid quadrupole Orbitrap mass spectrometer (Thermo Scientific™) in both ESI+ and ESI- modes. The spray voltages were set at 3.5 kV each, and the heated capillary temperature was maintained at 300 °C. For full mass scans, the parameters were as follows: resolution of 70,000, an auto gain control target set below 3 × 10^6^, maximum isolation time of 100 ms, and a scan range from 80 to 1200 m/z. For MS/MS scans, the parameters included a resolution of 17,500, an auto gain control target set below 1 × 10^5^, maximum isolation time of 50 ms, and normalized collision energies of 10, 30, and 60 v.

### Human corneal samples

Corneal samples were obtained from surgical procedures involving three DED patients for immunohistochemistry. The control group were intraocular tumor patients with normal corneal tissues identified by two senior ophthalmologists.

### Corneal epithelial cell culture and treatment

Human corneal epithelial cells (HCE-T, CL-0743) were provided by Procell Life Science & Technology Co., Ltd. The cells were cultured in DMEM/F12 medium supplemented with 10% FBS at 37 °C in a 5% CO_2_ incubator. To induce hyperosmotic conditions, HCE-T cells were exposed to 90 mmol/L NaCl, resulting in an osmolarity of 500 mOsm (measured by osmometer), and assessed after a 12-h incubation. When cells reached 80% confluence, GLS1 plasmid transfection was performed using the Endofectin in vitro DNA & siRNA Transfection Reagent (101000046, Polyplus-transfection S.A, Illkirch, France). Plasmid DNA was combined with the transfection reagent and incubated at room temperature for 10 min. Additionally, BPTES, a selective GLS1 inhibitor (Selleckchem, cat. S7024100-14b, 10 μM), was employed. Gene expression was evaluated 24–48 h post-transfection.

### High-throughput cytokine analysis

To assess the impact of cytokines or chemokines, a panel of cytokines, including those specified in the manufacturer’s instructions, was measured. The plate was analyzed using a Luminex 200 instrument (Luminex). Data acquisition and analysis were performed using Luminex xPONENT software.

### RNA-seq analysis

Total RNA extraction was carried out using the SMART-Seq® HT Kit. Paired-end libraries were constructed following the TruSeq® RNA Sample Preparation Guide (Illumina, USA) with the TruSeq® RNA Sample Preparation Kit (Illumina, USA). Subsequently, the libraries underwent purification and PCR-based enrichment to generate the final cDNA library. The quantification of purified libraries was conducted using the Qubit® 2.0 Fluorometer (Life Technologies, USA), and the library size and molar concentration were validated using the Agilent 2100 bioanalyzer (Agilent Technologies, USA). Clusters were formed via cBot, with library dilution to 10 pM, and sequencing was performed on the Illumina HiSeq Xten (Illumina, USA). Library preparation and sequencing procedures were carried out at Shanghai Biotechnology Corporation. Differential expression analysis was executed employing DESeq2 (Bioconductor). Resulting *p*-values underwent multiple testing correction using the Benjamini and Hochberg method. Genes exhibiting differential regulation were characterized by a log2FC > ± 1 and an adjusted *p* < 0.05. Heatmaps were generated utilizing Morpheus (Broad Institute).

### RT-qPCR analysis

Total RNA was extracted from cells using TRIzol and reversely transcribed to cDNA using ProtoScript® II First Strand cDNA Synthesis Kit (E6560S, NEB) according to the manufacturer’s instructions. RT-qPCR was conducted using PowerUp SYBR Green Master Mix (Applied Biosystems, Foster City, CA, USA) and performed using the CFX-96 (Bio-Rad). The cycling parameters were as follows: 10 min at 95 °C, 45 cycles of 10 s at 95 °C, 30 at 58 °C following the manufacturer’s instructions. Data were performed as fold induction relative to control group and the relative mRNA level of target gene was analyzed by the formula 2^–ΔCt^ (ΔCt = Ct^target^ - Ct^18S^). The primer sequences are shown in supplementary table.

### Western blot analysis

Cells were lysed in SDS buffer with protease and phosphatase inhibitors. The protein concentration was determined using a Pierce BCA protein assay kit (23225, Thermo Scientific, MA, USA). Equal amounts of protein were separated by SDS-polyacrylamide gel electrophoresis and then transferred onto nitrocellulose filter membranes. The membranes were blocked with 5% BSA and subsequently incubated with primary antibodies and secondary antibodies. Antigen-antibody complexes on the membranes were detected with an enhanced chemiluminescence kit from Thermo Scientific. Antibodies: glutaminase (ab156876), p65 (proteintech 10745), p-p65(proteintech), IκBalphaα (cell signaling technology 4812 s), p-IκBα (cell signaling technology 2859 s).

### Immunofluorescence staining

Sections were deparaffinized using xylene and rehydrated, followed by permeabilization with 0.2% Triton X-100 for 15 min and subsequent blocking with 5% bovine serum albumin in PBS for 1 h at room temperature. The sections were incubated overnight at 4 °C with primary antibodies against GLS1 (1:200, ab260047, Abcam) and MUC1 (1:100, ab109185, Abcam). Afterward, the sections underwent three washes with PBS and were then incubated with secondary antibodies. Staining using Alexa Fluor fluorophores was carried out for 1 h at room temperature. Subsequently, the sections were washed once more and mounted onto glass slides using Flouroshield Mounting Medium containing 4’,6-diamidino-2-phenylindole (Abcam, ab104139) for imaging via confocal fluorescence microscopy (Olympus, Tokyo, Japan).

### Immunohistochemical staining

Immunohistochemical staining was performed on paraffin sections following established protocols. Briefly, paraffin-embedded kidney sections were dewaxed, treated with 3% hydrogen peroxide, and subjected to microwave heating for antigen retrieval. The sections were then incubated with primary antibodies, specifically GLS1 (1:200, ab260047, Abcam), at 4 °C overnight. After rinsing with phosphate-buffered saline (PBS), the sections underwent incubation with horseradish peroxidase-streptavidin biotinylated secondary antibodies, followed by diaminobenzidine (DAB kit, Vector Laboratories, California, USA) for visualization.

### Single-Cell mRNA library preparation and sequencing

Surgical resected fresh corneal and conjunctiva samples were minced and enzymatically digested to obtain single-cell suspensions. The complementary deoxyribonucleic acid (cDNA) library was generated using a commercial 10x Genomics platform (10x Genomics, Pleasanton, CA, USA). Single-cell transcriptome amplification and library preparation were performed using the Single-Cell 3’ Library Kit v3 (10x Genomics) by Capitalbio Technology Corporation according to manufacturer’s instructions. Then, the libraries were pooled and sequenced across six lanes on an Illumina NovaSeq 6000 system (Illumina, Inc., San Diego, CA, USA).

### Pre-processing of scRNA-Seq data

The raw sequencing FASTQ files of rat DED model samples were aligned to themRatBN7.2 reference genome using the cellranger count function of CellRanger (10X Genomics, v5) to produce a gene expression matrix via the STAR algorithm. Then, the raw gene expression matrices of all samples were imported and processed by the Seurat^80^ R package (version 4.0.0) as previously reported. Low-quality cells were removed according to the following criteria: cells that had fewer than 2001 unique molecular identifiers (UMIs), more than 6000 or less than 301 expressed genes, or over 25% of UMIs derived from the mitochondrial genome as described previously. Included genes were expressed in at least ten cells in a sample. We removed potential cell doublets using the DoubletFinder^81^ R package. The single cell transcriptome expression matrices of the remaining high-quality cells were integrated with the“RunFastMNN” function of SeuratWrappers package, normalized to the total cellular UMI count, and scaled (scale.factor = 1e4) by regressing out the total cellular UMI counts and percentage of mitochondrial genes. Then, we selected highly variable genes (HVGs) for principal component analysis (PCA), and the top 30 significant principal components (PCs) were selected for Uniform Manifold Approximation and Projection (UMAP) dimension reduction and visualization of gene expression.

### Cell type abundance estimation

To characterize the tissue distribution of meta-clusters, odds ratios (OR) were calculated and used to indicate preferences as previously reported^82^. Then Fisher’s exact test was applied on this contingency table, thus OR and corresponding *p*-value could be obtained. *P*-values were adjusted using the BH method implemented in the R function p.adjust. We found that all ORs > 1.5 or ORs < 0.5 had adjusted *p*-values < 1e-10. Hence, a higher OR with a value > 1.5 indicated that meta-cluster i was more preferred to distribute in tissue j, a lower OR with a value < 0.5 indicated that meta-cluster i was preferred not to distribute in tissue j.

### Trajectory analysis

To explore the potential differentiation routines between corneal epithelial cells subtypes, we performed the trajectory analysis via Slingshot^83^ package as previously reported. For Slingshot trajectory inference analysis, the Seurat object was imported into the Slingshot and PCA-based dimension reduction was performed with differentially expressed genes, followed by two-dimensional visualization with UMAP.

### Statistical analysis

Data were analyzed with GraphPad Prism 9.0. Statistical analysis was performed using one-way ANOVA for multiple experimental groups. Data are reported as the mean values with error bars showing the standard error of the mean (SEM). When ANOVA showed a significant difference, post hoc analysis between group means was examined by Tukey’s or uncorrected Fisher’s LSD multiple-comparison test^84^. *p* < 0.05 was considered statistically significant.

## Supplementary information

Supplementary Materials

## Data Availability

All datasets generated during and analyzed during the current study are available from the corresponding author on reasonable request. The transcriptomic data of L-glutamine treated group, D-glutamine treated group and DED group in an in vitro dry eye model has been deposited in the NCBI Sequence Read Archive (SRA) database (accession number: PRJNA1198298). The data of scRNA-seq in this paper have been deposited in the Genome Sequence Archive (GSA) under accession number OMIX008264 and the processed data can be accessed in https://ngdc.cncb.ac.cn/omix.
