# Supplementary material for: Identification of glutamine as a potential therapeutic target in dry eye disease
Source: Signal Transduct Target Ther. 2025 Jan 22;10:27. doi: 10.1038/s41392-024-02119-1 (PMC11751114; doi:10.1038/s41392-024-02119-1)
Supplement: Supplementary file 1 — Supplementary Materials [file 41392_2024_2119_MOESM1_ESM.docx]

Supplementary Materials for

Identification of glutamine as a potential therapeutic target in dry eye disease

Xiaoniao Chen, Chuyue Zhang, Fei Peng, Lingling Wu, Deyi Zhuo, Liqiang Wang, Min Zhang, Zhaohui Li, Lei Tian, Ying Jie, Yifei Huang, Xinji Yang, Xiaoqi Li, Fengyang Lei, Yu Cheng

Correspondence to: X. Chen (1525980867@qq.com)

**This PDF file includes:**

Figs. S1 to S3

Tables S1 to S4.

**Other Supplementary Materials for this manuscript include the following:**

Fig. S4 Uncropped western blots

**Table S1**. Metabolite identity putatively assigned in rat cornea tissues by positive ion MALDI-TOF/TOF MS and LC/MS-MS.

| Parent ion | m/z | Structurally specific MS/MS peaks | Assignment | formula | Identity methods |
| --- | --- | --- | --- | --- | --- |
| [M+H]^+^ | 147.3 | 56, 84, 102, 130 | D-Glutamine | C5H10N2O3 | LC/MS-MS |
| [M+H]^+^ | 165.3 | 53, 95, 119, 123, 147 | 2-Hydroxycinnamic acid | C9H8O3 | LC/MS-MS |
| [M+H]^+^ | 286.4 | 59, 142, 256 | Myristoylglycine | C16H32NO3 | MALDI-TOF/TOF MS |
| [M+H]^+^ | 302.3 | 240, 272, 283 | Sphinganine | C18H40NO2 | MALDI-TOF/TOF MS |
| [M+H]^+^ | 666.5 | 250, 440, 650 | NADH | C21H30N7O14P2 | MALDI-TOF/TOF MS |
| [M+H]^+^ | 862.8 | 86, 250, 666, 804 | PC(42:6) | C50H89NO8P | MALDI-TOF/TOF MS |

**Fig. S1**


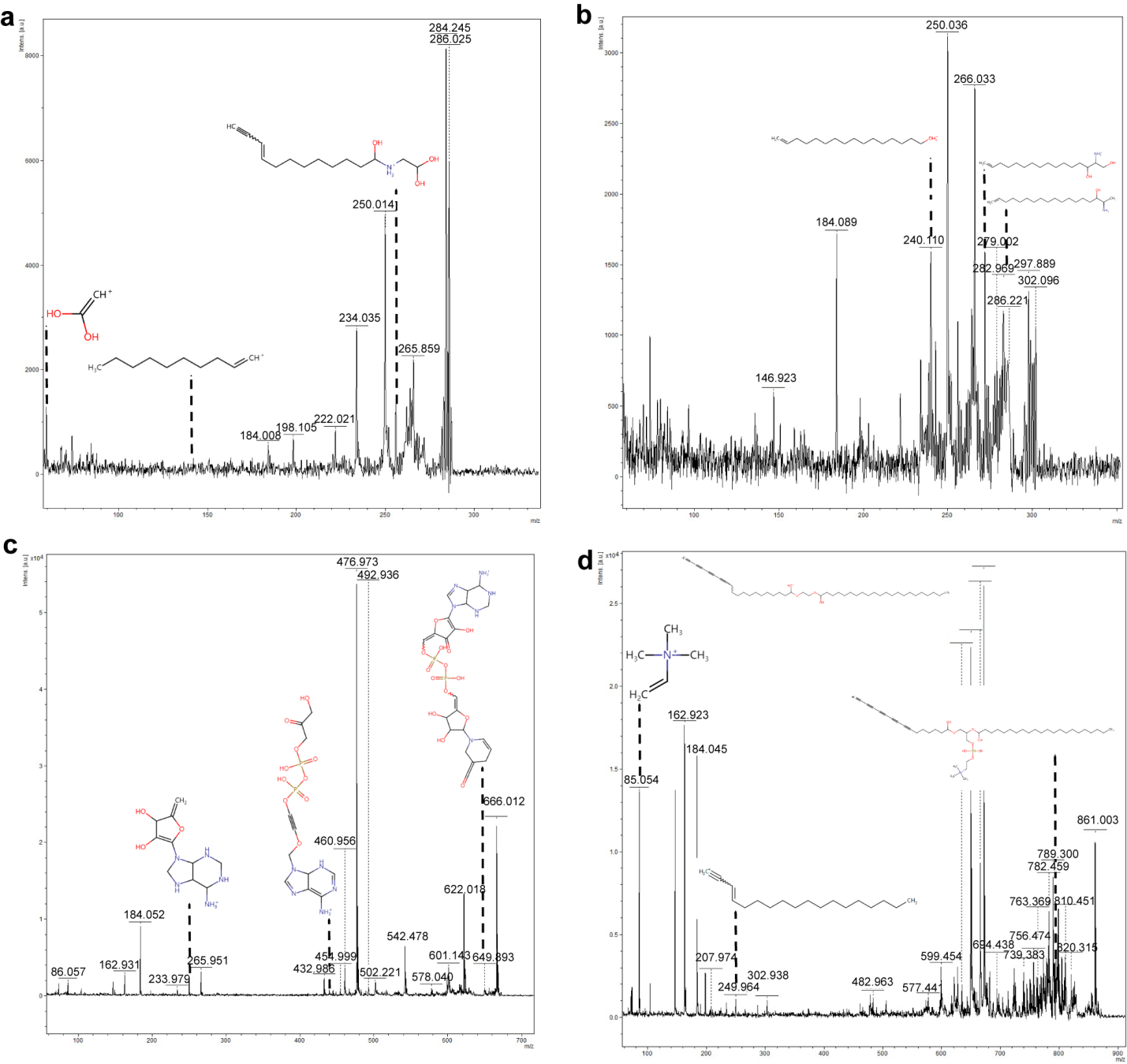


**Fig. S1.** *In situ* MALDI-MS/MS spectra in positive mode following parent ions of representative metabolites and the assignment of the fragment ions. **a** m/z 286; **b** m/z 302; **c** m/z 666; **d** m/z 862 .

**Fig. S2**


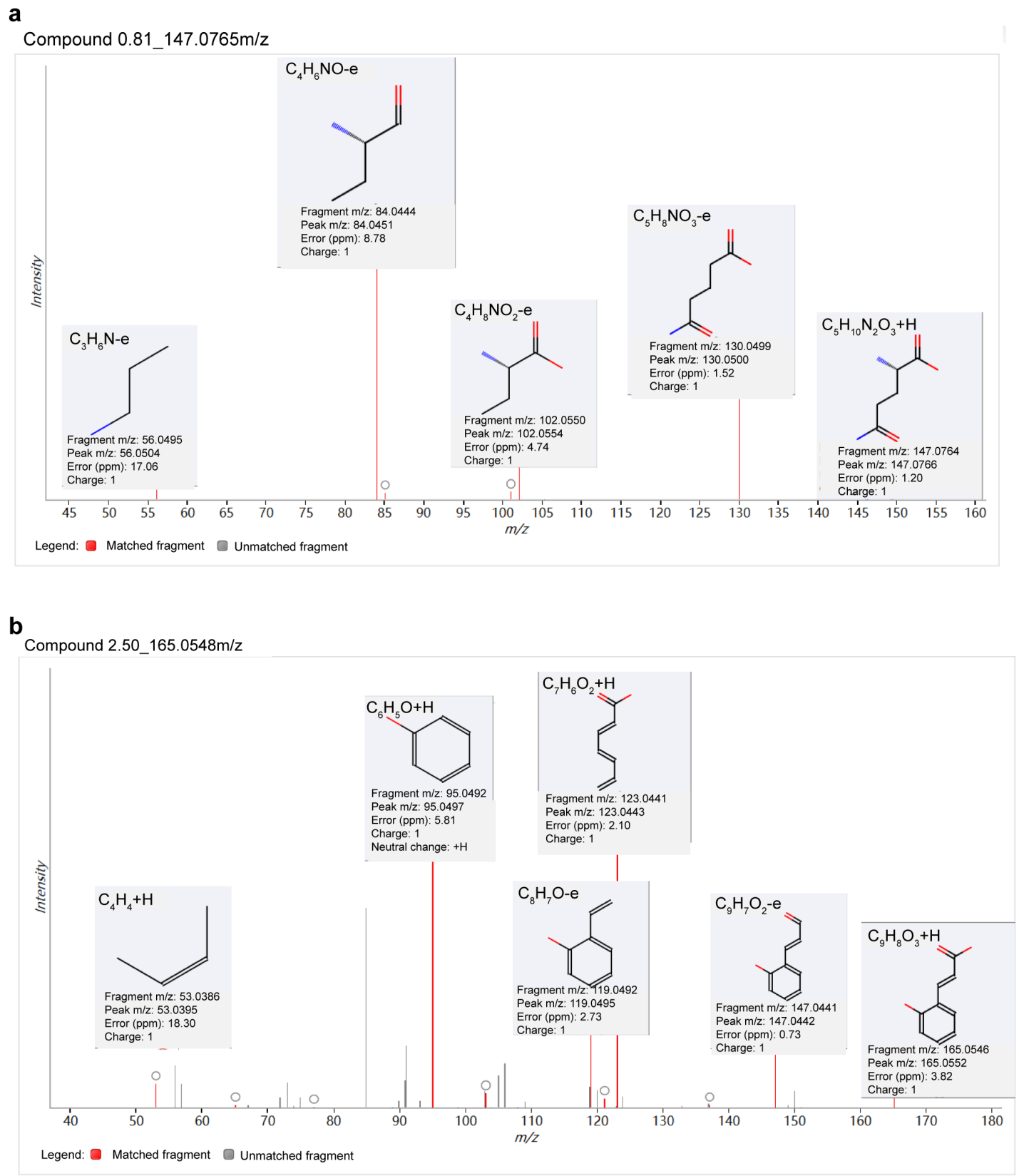


**Fig. S2**. LCMS-MS/MS spectra in positive mode following parent ions of representative metabolite glutamine (a) and 2-Hydroxycinnamic acid (b) and the assignment of the fragment ions. a m/z 147; b m/z 165.

**Fig. S3**


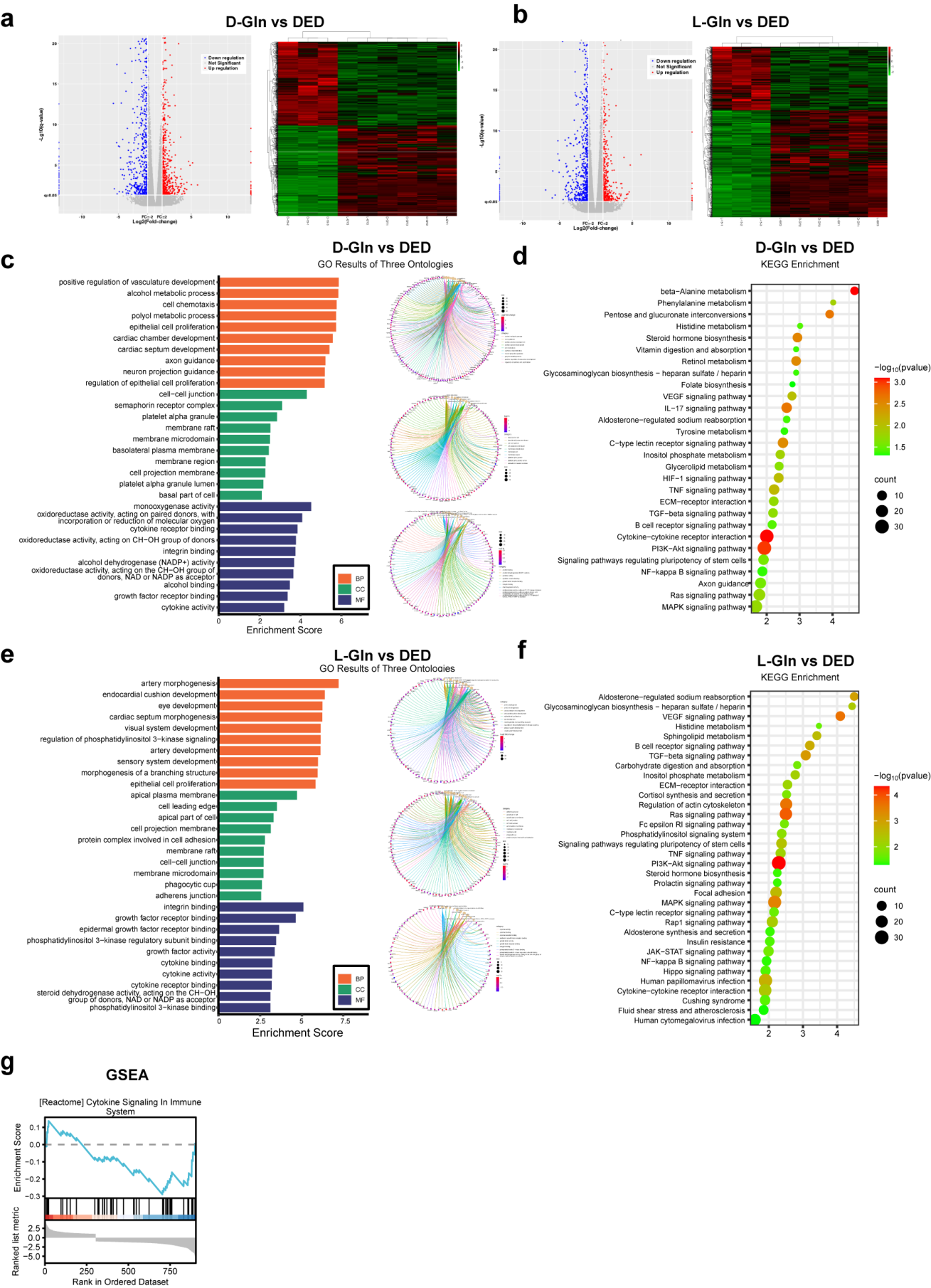


**Fig. S3**. The transcriptomic data of L-glutamine treated group, D-glutamine treated group and DED group in an *in vitro* dry eye model.

| Table S2. Information of clinical patients | | | | | | | | | | |
| --- | --- | --- | --- | --- | --- | --- | --- | --- | --- | --- |
| Number | Dry Eye Disease status of patients | Number | Gender | Age | Biospecimen type | Clinical characteristics of patients | Clinical diagnosis of patients | Eyeball protrusion | Collection mechanism |  |
| Con 1 | Control | Y5887840 | Female | 69 | Solid tissue | Intraocular tumor | Bulbar metastases of meningioma (OD) | OD:13mm; OS:12mm, IOD：108mm | surgery |  |
| Con 2 | Control | Y4402427 | Male | 52 | Solid tissue | Intraocular tumor, Choriomelanoma | Choriomelanoma (OD) | OD:13mm; OS:13mm, IOD：105mm | surgery |  |
| Con 3 | Control | H0465700 | Female | 2 | Solid tissue | Intraocular tumor, Retinoblastoma | Retinoblastoma（OD） | OD:14mm; OS:13mm, IOD：100mm | surgery |  |
| Patient 1 | Dry eye, Hypophasis | Y7313571 | Female | 69 | Solid tissue | Intraorbital tumor | Intraorbital tumor (OD) | OD:18mm; OS:12mm, IOD：110mm | surgery |  |
| Patient 2 | Dry eye, Hypophasis | H0480245 | Female | 30 | Solid tissue | Intraorbital tumor | Intraorbital tumor (OD) | OD:17mm; OS:17mm, IOD：112mm | surgery |  |
| Patient 3 | Dry eye, Hypophasis | 3060073 | Male | 62 | Solid tissue | Intraorbital tumor | Lacrimal mucous epidermoid carcinoma (OS) | OD:12mm; OS:17mm, IOD：98mm | surgery |  |

| **Table S3**. Upplemental table S1 The top 5 differentially expressed genes (DEGs) in 13 main cell types | | | | | | | |
| --- | --- | --- | --- | --- | --- | --- | --- |
|  | p_val | avg_log2FC | pct.1 | pct.2 | p_val_adj | cluster | gene |
| 1 | 0 | 4.424846056 | 0.777 | 0.023 | 0 | Endo_Lyve1 | Mmrn1 |
| 2 | 0 | 3.352995956 | 0.734 | 0.015 | 0 | Endo_Lyve1 | Prox1 |
| 3 | 4.81E-271 | 4.772974211 | 0.798 | 0.038 | 9.42E-267 | Endo_Lyve1 | Reln |
| 4 | 9.19E-198 | 3.903849904 | 0.809 | 0.058 | 1.80E-193 | Endo_Lyve1 | Lyve1 |
| 5 | 4.80E-120 | 6.804052642 | 0.745 | 0.087 | 9.40E-116 | Endo_Lyve1 | Ccl21 |
| 6 | 0 | 4.279329651 | 0.818 | 0.079 | 0 | Endo_Emcn | Flt1 |
| 7 | 0 | 3.380410443 | 0.703 | 0.037 | 0 | Endo_Emcn | Adgrl4 |
| 8 | 0 | 3.196342233 | 0.654 | 0.021 | 0 | Endo_Emcn | Cyyr1 |
| 9 | 0 | 3.092649405 | 0.677 | 0.038 | 0 | Endo_Emcn | Emcn |
| 10 | 1.96E-77 | 3.488915924 | 0.303 | 0.056 | 3.83E-73 | Endo_Emcn | Fabp4 |
| 11 | 7.36E-60 | 2.515960521 | 0.917 | 0.067 | 1.44E-55 | Endo_Flt1 | Adgrl4 |
| 12 | 3.20E-51 | 2.586376048 | 0.958 | 0.089 | 6.26E-47 | Endo_Flt1 | Apold1 |
| 13 | 8.94E-49 | 4.004393907 | 1 | 0.113 | 1.75E-44 | Endo_Flt1 | Flt1 |
| 14 | 1.59E-29 | 3.879323039 | 0.625 | 0.067 | 3.11E-25 | Endo_Flt1 | Fabp4 |
| 15 | 1.14E-27 | 2.557754637 | 0.958 | 0.18 | 2.24E-23 | Endo_Flt1 | Adamts9 |
| 16 | 7.82E-293 | 3.841383614 | 0.811 | 0.166 | 1.53E-288 | Pericyte | Myh11 |
| 17 | 5.65E-286 | 3.794184899 | 0.776 | 0.151 | 1.11E-281 | Pericyte | Tagln |
| 18 | 1.05E-238 | 3.183773991 | 0.797 | 0.203 | 2.06E-234 | Pericyte | RGD1564664 |
| 19 | 9.52E-194 | 3.296005547 | 0.833 | 0.299 | 1.86E-189 | Pericyte | Tpm2 |
| 20 | 5.91E-152 | 3.291521721 | 0.651 | 0.196 | 1.16E-147 | Pericyte | Dmd |
| 21 | 0 | 4.052320389 | 0.978 | 0.082 | 0 | Corneal_Fib_Kera | Angptl7 |
| 22 | 0 | 3.933098111 | 0.995 | 0.22 | 0 | Corneal_Fib_Kera | Apod |
| 23 | 0 | 3.922730861 | 0.994 | 0.247 | 0 | Corneal_Fib_Kera | Chsy3 |
| 24 | 0 | 3.90459727 | 0.996 | 0.357 | 0 | Corneal_Fib_Kera | Kera |
| 25 | 0 | 3.724589921 | 0.995 | 0.27 | 0 | Corneal_Fib_Kera | A2m |
| 26 | 0 | 3.075230851 | 0.99 | 0.784 | 0 | Fib_Col3a1 | Col3a1 |
| 27 | 0 | 2.993374206 | 0.918 | 0.427 | 0 | Fib_Col3a1 | Gfpt2 |
| 28 | 0 | 2.797012362 | 0.998 | 0.885 | 0 | Fib_Col3a1 | Gsn |
| 29 | 0 | 2.772166435 | 0.828 | 0.288 | 0 | Fib_Col3a1 | Has1 |
| 30 | 0 | 2.712008063 | 0.856 | 0.156 | 0 | Fib_Col3a1 | Adgrd1 |
| 31 | 0 | 5.360918682 | 0.98 | 0.086 | 0 | Fib_Col9a2 | Ucma |
| 32 | 0 | 4.34184127 | 0.96 | 0.052 | 0 | Fib_Col9a2 | Cnmd |
| 33 | 0 | 4.212779228 | 0.886 | 0.059 | 0 | Fib_Col9a2 | Snorc |
| 34 | 0 | 4.067203744 | 0.955 | 0.05 | 0 | Fib_Col9a2 | Col9a2 |
| 35 | 7.76E-274 | 5.087190891 | 0.95 | 0.133 | 1.52E-269 | Fib_Col9a2 | Cytl1 |
| 36 | 0 | 3.758519435 | 0.596 | 0.021 | 0 | Schwann_cell_Cdh19 | Grik2 |
| 37 | 0 | 3.472482029 | 0.588 | 0.006 | 0 | Schwann_cell_Cdh19 | Cdh19 |
| 38 | 0 | 3.317330673 | 0.581 | 0.014 | 0 | Schwann_cell_Cdh19 | Il1rapl1 |
| 39 | 1.32E-284 | 3.917394805 | 0.529 | 0.019 | 2.59E-280 | Schwann_cell_Cdh19 | Scn7a |
| 40 | 3.27E-108 | 5.233604528 | 0.36 | 0.026 | 6.40E-104 | Schwann_cell_Cdh19 | Mpz |
| 41 | 2.24E-184 | 3.974319967 | 0.584 | 0.024 | 4.39E-180 | Melanocytes | Clcnkb |
| 42 | 1.14E-91 | 3.485241171 | 0.364 | 0.019 | 2.23E-87 | Melanocytes | Coch |
| 43 | 1.33E-79 | 4.847155507 | 0.584 | 0.064 | 2.60E-75 | Melanocytes | Gp2 |
| 44 | 3.17E-43 | 3.324076704 | 0.844 | 0.307 | 6.20E-39 | Melanocytes | Slc12a2 |
| 45 | 1.12E-14 | 3.778886135 | 0.87 | 0.776 | 2.19E-10 | Melanocytes | AABR07000398.1 |
| 46 | 0 | 3.884146832 | 0.918 | 0.85 | 0 | Corneal_Epi | Krt12 |
| 47 | 3.60E-204 | 3.157523308 | 0.707 | 0.531 | 7.04E-200 | Corneal_Epi | Lypd2 |
| 48 | 1.19E-142 | 2.894156853 | 0.498 | 0.247 | 2.33E-138 | Corneal_Epi | Aqp5 |
| 49 | 8.69E-17 | 4.21949819 | 0.185 | 0.116 | 1.70E-12 | Corneal_Epi | Hba-a1 |
| 50 | 2.14E-07 | 4.074363936 | 0.152 | 0.114 | 0.004198458 | Corneal_Epi | LOC100134871 |
| 51 | 0 | 2.920550262 | 0.968 | 0.669 | 0 | Conjunctiva_Epi | Krt15 |
| 52 | 0 | 2.85121004 | 0.809 | 0.246 | 0 | Conjunctiva_Epi | Krt13 |
| 53 | 2.55E-248 | 2.962522043 | 0.645 | 0.194 | 4.99E-244 | Conjunctiva_Epi | Plac8 |
| 54 | 4.38E-201 | 3.651622738 | 0.715 | 0.324 | 8.57E-197 | Conjunctiva_Epi | MGC105649 |
| 55 | 2.09E-174 | 3.190238304 | 0.534 | 0.159 | 4.09E-170 | Conjunctiva_Epi | S100g |
| 56 | 0 | 4.458580066 | 0.855 | 0.024 | 0 | T_cell | Camk4 |
| 57 | 0 | 3.534209456 | 0.581 | 0.005 | 0 | T_cell | Icos |
| 58 | 1.74E-204 | 4.660317511 | 0.758 | 0.032 | 3.41E-200 | T_cell | Ikzf3 |
| 59 | 1.48E-183 | 4.167758911 | 0.919 | 0.055 | 2.89E-179 | T_cell | Ptprc |
| 60 | 1.59E-96 | 5.487846459 | 0.403 | 0.019 | 3.12E-92 | T_cell | Ccl5 |
| 61 | 0 | 4.721122585 | 0.903 | 0.086 | 0 | Mono/Mac | Lyz2 |
| 62 | 2.80E-159 | 5.124946279 | 0.773 | 0.229 | 5.49E-155 | Mono/Mac | Cxcl2 |
| 63 | 7.29E-144 | 4.837190647 | 0.553 | 0.107 | 1.43E-139 | Mono/Mac | RT1-Db1 |
| 64 | 2.52E-137 | 4.906954844 | 0.553 | 0.113 | 4.93E-133 | Mono/Mac | RT1-Da |
| 65 | 2.23E-129 | 5.159781877 | 0.638 | 0.168 | 4.37E-125 | Mono/Mac | Cd74 |

**Table S4**. The top 25 differentially expressed genes (DEGs) in 5 corneal epithelial cells subtypes

|  | p_val | avg_log2FC | pct.1 | pct.2 | p_val_adj | cluster | gene |
| --- | --- | --- | --- | --- | --- | --- | --- |
| 1 | 0 | 2.371611279 | 0.945 | 0.385 | 0 | Epi1 | Aqp5 |
| 2 | 0 | 2.29394276 | 0.997 | 0.924 | 0 | Epi1 | Krt12 |
| 3 | 0 | 2.222079777 | 0.929 | 0.174 | 0 | Epi1 | Dsc3 |
| 4 | 0 | 2.195657905 | 0.97 | 0.676 | 0 | Epi1 | Lypd2 |
| 5 | 0 | 2.170671375 | 0.811 | 0.067 | 0 | Epi1 | Dync1i1 |
| 6 | 0 | 1.988060843 | 0.984 | 0.487 | 0 | Epi1 | Dsp |
| 7 | 0 | 1.944516795 | 0.692 | 0.025 | 0 | Epi1 | Kcnj3 |
| 8 | 0 | 1.879299954 | 0.946 | 0.436 | 0 | Epi1 | Mgarp |
| 9 | 0 | 1.794202479 | 0.878 | 0.119 | 0 | Epi1 | Fam83a |
| 10 | 0 | 1.791573674 | 0.865 | 0.076 | 0 | Epi1 | Erich5 |
| 11 | 0 | 1.789140285 | 0.684 | 0.085 | 0 | Epi1 | Trpm3 |
| 12 | 0 | 1.750329621 | 0.995 | 0.864 | 0 | Epi1 | Krt5 |
| 13 | 0 | 1.720908172 | 0.997 | 0.923 | 0 | Epi1 | Aldh3a1 |
| 14 | 0 | 1.720186373 | 0.789 | 0.047 | 0 | Epi1 | Dsg1 |
| 15 | 0 | 1.710535929 | 0.693 | 0.062 | 0 | Epi1 | Thsd7a |
| 16 | 0 | 1.698948831 | 0.787 | 0.057 | 0 | Epi1 | Ctnnd2 |
| 17 | 0 | 1.691129182 | 0.881 | 0.214 | 0 | Epi1 | Dsg3 |
| 18 | 0 | 1.690093627 | 0.775 | 0.077 | 0 | Epi1 | Samd12 |
| 19 | 0 | 1.678494486 | 0.807 | 0.1 | 0 | Epi1 | Meis2 |
| 20 | 0 | 1.650579714 | 0.849 | 0.182 | 0 | Epi1 | Oxr1 |
| 21 | 0 | 1.63913858 | 0.916 | 0.225 | 0 | Epi1 | Psme4 |
| 22 | 2.21E-280 | 1.664475881 | 0.943 | 0.428 | 4.33E-276 | Epi1 | Cst6 |
| 23 | 4.86E-269 | 2.029280683 | 0.752 | 0.199 | 9.52E-265 | Epi1 | Col17a1 |
| 24 | 9.05E-259 | 1.702323588 | 0.971 | 0.558 | 1.77E-254 | Epi1 | Tkt |
| 25 | 2.26E-251 | 1.977961435 | 0.886 | 0.363 | 4.43E-247 | Epi1 | Slurp1 |
| 26 | 4.17E-105 | 1.397552637 | 0.988 | 0.933 | 8.16E-101 | Epi2 | Krt12 |
| 27 | 1.01E-102 | 1.548735913 | 0.944 | 0.713 | 1.98E-98 | Epi2 | Lypd2 |
| 28 | 1.41E-100 | 1.268641696 | 0.983 | 0.933 | 2.77E-96 | Epi2 | Aldh3a1 |
| 29 | 4.87E-82 | 1.104222012 | 0.986 | 0.88 | 9.53E-78 | Epi2 | Krt5 |
| 30 | 4.74E-74 | 1.325724416 | 0.924 | 0.786 | 9.27E-70 | Epi2 | Txn1 |
| 31 | 4.49E-69 | 1.665795257 | 0.671 | 0.37 | 8.79E-65 | Epi2 | Dapl1 |
| 32 | 1.04E-63 | 1.462381843 | 0.764 | 0.489 | 2.04E-59 | Epi2 | Gpha2 |
| 33 | 1.18E-63 | 1.212049563 | 0.793 | 0.511 | 2.31E-59 | Epi2 | Mgarp |
| 34 | 1.36E-53 | 1.218097613 | 0.806 | 0.623 | 2.66E-49 | Epi2 | Tkt |
| 35 | 1.01E-50 | 1.63705955 | 0.676 | 0.445 | 1.98E-46 | Epi2 | Slurp1 |
| 36 | 2.24E-41 | 1.197230463 | 0.674 | 0.478 | 4.38E-37 | Epi2 | Ptgr1 |
| 37 | 6.85E-36 | 1.061458164 | 0.667 | 0.519 | 1.34E-31 | Epi2 | Atp5if1 |
| 38 | 3.32E-33 | 1.051725168 | 0.684 | 0.515 | 6.51E-29 | Epi2 | Cst6 |
| 39 | 3.86E-30 | 1.079760805 | 0.669 | 0.479 | 7.55E-26 | Epi2 | Aqp5 |
| 40 | 2.40E-22 | 1.092617494 | 0.55 | 0.442 | 4.70E-18 | Epi2 | Dbi |
| 41 | 1.15E-20 | 1.083814435 | 0.426 | 0.269 | 2.26E-16 | Epi2 | LOC102551453 |
| 42 | 6.95E-20 | 0.948309092 | 0.589 | 0.461 | 1.36E-15 | Epi2 | Mal |
| 43 | 2.62E-13 | 1.140827939 | 0.32 | 0.216 | 5.12E-09 | Epi2 | Fam25a |
| 44 | 8.41E-12 | 1.112558606 | 0.442 | 0.373 | 1.65E-07 | Epi2 | Csrp2 |
| 45 | 2.18E-09 | 0.966549126 | 0.314 | 0.231 | 4.27E-05 | Epi2 | Clic3 |
| 46 | 2.91E-09 | 1.129928869 | 0.269 | 0.181 | 5.71E-05 | Epi2 | Spink13 |
| 47 | 4.21E-07 | 0.935035326 | 0.207 | 0.14 | 0.008247499 | Epi2 | LOC100912228 |
| 48 | 2.19E-05 | 1.046969769 | 0.227 | 0.175 | 0.428997815 | Epi2 | Slurp2 |
| 49 | 2.81E-05 | 0.99838951 | 0.324 | 0.275 | 0.549717869 | Epi2 | Cxcl14 |
| 50 | 0.000498271 | 1.020649476 | 0.281 | 0.247 | 1 | Epi2 | Sbsn |
| 51 | 2.20E-172 | 1.999836911 | 0.975 | 0.785 | 4.31E-168 | Epi3 | Krt15 |
| 52 | 2.32E-123 | 2.225203114 | 0.767 | 0.419 | 4.55E-119 | Epi3 | Krt13 |
| 53 | 4.12E-92 | 1.42858666 | 0.964 | 0.869 | 8.07E-88 | Epi3 | S100a11 |
| 54 | 1.64E-87 | 2.17023371 | 0.787 | 0.612 | 3.21E-83 | Epi3 | Lgals7 |
| 55 | 2.22E-79 | 1.979547926 | 0.637 | 0.349 | 4.34E-75 | Epi3 | Wfdc21 |
| 56 | 1.70E-65 | 2.069232271 | 0.533 | 0.276 | 3.33E-61 | Epi3 | Ccl6 |
| 57 | 1.32E-63 | 2.078248691 | 0.423 | 0.168 | 2.59E-59 | Epi3 | Krt4 |
| 58 | 5.12E-52 | 1.842005984 | 0.307 | 0.102 | 1.00E-47 | Epi3 | Serpinb10 |
| 59 | 7.09E-52 | 1.141091009 | 0.872 | 0.75 | 1.39E-47 | Epi3 | AABR07028446.1 |
| 60 | 1.98E-51 | 1.097178989 | 0.805 | 0.649 | 3.88E-47 | Epi3 | Calml3 |
| 61 | 2.34E-40 | 1.05740667 | 0.828 | 0.759 | 4.58E-36 | Epi3 | S100a10 |
| 62 | 7.28E-34 | 1.769138578 | 0.458 | 0.291 | 1.43E-29 | Epi3 | Plac8 |
| 63 | 9.01E-30 | 1.158878279 | 0.624 | 0.547 | 1.76E-25 | Epi3 | Taldo1 |
| 64 | 7.39E-24 | 1.437980466 | 0.418 | 0.289 | 1.45E-19 | Epi3 | Pdzk1ip1 |
| 65 | 1.38E-19 | 1.199085696 | 0.523 | 0.423 | 2.71E-15 | Epi3 | MGC105649 |
| 66 | 1.37E-17 | 1.687621593 | 0.383 | 0.29 | 2.68E-13 | Epi3 | Defb1 |
| 67 | 2.41E-15 | 1.76522226 | 0.409 | 0.308 | 4.72E-11 | Epi3 | S100g |
| 68 | 3.26E-10 | 1.158957865 | 0.192 | 0.112 | 6.39E-06 | Epi3 | Cyp4a8 |
| 69 | 1.74E-07 | 0.991735933 | 0.43 | 0.425 | 0.003408402 | Epi3 | Gltp |
| 70 | 7.37E-07 | 1.258569579 | 0.233 | 0.177 | 0.014426138 | Epi3 | Wfdc2 |
| 71 | 6.05E-06 | 1.546541673 | 0.356 | 0.332 | 0.118425289 | Epi3 | Ifitm1 |
| 72 | 1.62E-05 | 1.170249353 | 0.177 | 0.129 | 0.316957142 | Epi3 | Chmp4c |
| 73 | 2.02E-05 | 1.316791522 | 0.3 | 0.279 | 0.396001011 | Epi3 | Gchfr |
| 74 | 4.88E-05 | 1.082317634 | 0.197 | 0.15 | 0.954857403 | Epi3 | Tspan8 |
| 75 | 0.000110139 | 1.020143471 | 0.309 | 0.288 | 1 | Epi3 | Krt8 |
| 76 | 2.67E-156 | 1.29376376 | 0.964 | 0.92 | 5.23E-152 | Epi4 | Col1a1 |
| 77 | 1.58E-122 | 1.076113984 | 0.903 | 0.824 | 3.10E-118 | Epi4 | Dcn |
| 78 | 1.22E-62 | 0.922906034 | 0.827 | 0.747 | 2.39E-58 | Epi4 | Col3a1 |
| 79 | 3.00E-55 | 1.059278022 | 0.681 | 0.724 | 5.88E-51 | Epi4 | AABR07000398.1 |
| 80 | 2.89E-46 | 1.111332265 | 0.698 | 0.694 | 5.65E-42 | Epi4 | Sparc |
| 81 | 4.84E-37 | 1.398969425 | 0.587 | 0.594 | 9.48E-33 | Epi4 | Igfbp5 |
| 82 | 5.23E-37 | 1.274022268 | 0.518 | 0.499 | 1.02E-32 | Epi4 | Lum |
| 83 | 5.26E-37 | 0.859874693 | 0.539 | 0.462 | 1.03E-32 | Epi4 | Vim |
| 84 | 3.50E-30 | 0.944501175 | 0.646 | 0.592 | 6.85E-26 | Epi4 | Mgp |
| 85 | 2.65E-29 | 1.10153494 | 0.493 | 0.466 | 5.19E-25 | Epi4 | Fn1 |
| 86 | 9.31E-18 | 0.861651753 | 0.471 | 0.471 | 1.82E-13 | Epi4 | Fgl2 |
| 87 | 1.80E-17 | 0.959771009 | 0.545 | 0.632 | 3.52E-13 | Epi4 | Capn13 |
| 88 | 5.64E-16 | 1.064047509 | 0.372 | 0.348 | 1.10E-11 | Epi4 | Fstl1 |
| 89 | 3.87E-14 | 1.029309234 | 0.629 | 0.734 | 7.57E-10 | Epi4 | Igfbp2 |
| 90 | 7.35E-12 | 1.169130162 | 0.419 | 0.449 | 1.44E-07 | Epi4 | Col6a2 |
| 91 | 2.87E-10 | 1.061746074 | 0.362 | 0.366 | 5.62E-06 | Epi4 | Aebp1 |
| 92 | 6.61E-09 | 1.15786497 | 0.15 | 0.108 | 0.000129328 | Epi4 | Col5a1 |
| 93 | 1.85E-07 | 1.012838181 | 0.34 | 0.35 | 0.003626714 | Epi4 | Dpt |
| 94 | 3.85E-06 | 1.383745293 | 0.19 | 0.165 | 0.075383769 | Epi4 | Col5a2 |
| 95 | 8.07E-06 | 1.061894871 | 0.13 | 0.2 | 0.157950679 | Epi4 | Matn4 |
| 96 | 1.30E-05 | 1.061137215 | 0.175 | 0.264 | 0.255413691 | Epi4 | Ccl11 |
| 97 | 2.57E-05 | 1.474106638 | 0.33 | 0.364 | 0.502336937 | Epi4 | AABR07068316.1 |
| 98 | 8.36E-05 | 0.909294769 | 0.316 | 0.335 | 1 | Epi4 | Serping1 |
| 99 | 0.000322894 | 1.309282351 | 0.249 | 0.37 | 1 | Epi4 | A2m |
| 100 | 0.006726231 | 1.152729715 | 0.15 | 0.14 | 1 | Epi4 | Col6a1 |
| 101 | 0 | 1.88828989 | 0.707 | 0.12 | 0 | Epi5 | LOC100134871 |
| 102 | 0 | 1.563939301 | 0.648 | 0.058 | 0 | Epi5 | Cfd |
| 103 | 0 | 1.501361198 | 0.643 | 0.065 | 0 | Epi5 | C1qa |
| 104 | 0 | 1.451479797 | 0.642 | 0.068 | 0 | Epi5 | C1qb |
| 105 | 0 | 1.348163884 | 0.647 | 0.064 | 0 | Epi5 | Hba-a2 |
| 106 | 0 | 1.330396669 | 0.606 | 0.035 | 0 | Epi5 | Mylpf |
| 107 | 0 | 1.28181098 | 0.628 | 0.065 | 0 | Epi5 | Ccl21 |
| 108 | 0 | 1.277403716 | 0.635 | 0.06 | 0 | Epi5 | Tnnc2 |
| 109 | 0 | 1.254981007 | 0.629 | 0.073 | 0 | Epi5 | Lyz2 |
| 110 | 0 | 1.229336367 | 0.59 | 0.036 | 0 | Epi5 | Pf4 |
| 111 | 0 | 1.223413664 | 0.664 | 0.089 | 0 | Epi5 | RT1-Da |
| 112 | 0 | 1.170489963 | 0.649 | 0.081 | 0 | Epi5 | RT1-Db1 |
| 113 | 0 | 1.16458319 | 0.635 | 0.074 | 0 | Epi5 | C1qc |
| 114 | 0 | 1.144198844 | 0.624 | 0.074 | 0 | Epi5 | Bcl2a1 |
| 115 | 3.50E-283 | 1.413972951 | 0.659 | 0.113 | 6.84E-279 | Epi5 | S100a4 |
| 116 | 1.93E-262 | 1.430924105 | 0.726 | 0.145 | 3.78E-258 | Epi5 | Hba-a1 |
| 117 | 4.67E-259 | 1.174684536 | 0.711 | 0.139 | 9.14E-255 | Epi5 | Ccl7 |
| 118 | 8.36E-259 | 1.230330014 | 0.704 | 0.146 | 1.64E-254 | Epi5 | Cd74 |
| 119 | 5.81E-215 | 1.187241769 | 0.939 | 0.652 | 1.14E-210 | Epi5 | Ccl2 |
| 120 | 2.28E-202 | 1.446919537 | 0.782 | 0.325 | 4.47E-198 | Epi5 | Timp1 |
| 121 | 7.01E-201 | 1.386258292 | 0.895 | 0.641 | 1.37E-196 | Epi5 | S100a6 |
| 122 | 3.09E-195 | 1.158331249 | 0.667 | 0.167 | 6.06E-191 | Epi5 | Rarres2 |
| 123 | 1.50E-190 | 1.378686518 | 0.806 | 0.406 | 2.94E-186 | Epi5 | Crip1 |
| 124 | 5.23E-159 | 1.205489615 | 0.756 | 0.316 | 1.02E-154 | Epi5 | Mt1 |
| 125 | 2.71E-129 | 1.18521528 | 0.689 | 0.325 | 5.30E-125 | Epi5 | Hspa8 |
|  |  |  |  |  |  |  |  |

**Fig. S4**

Fig. 5b


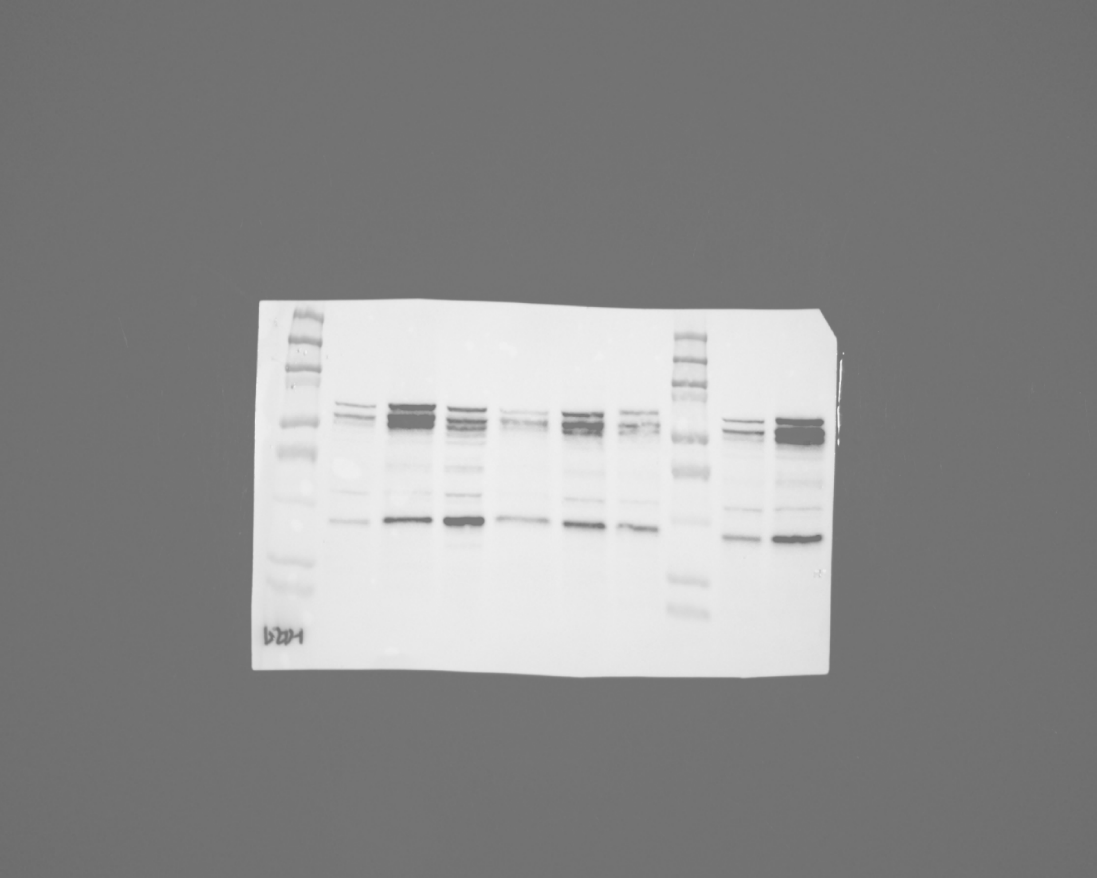

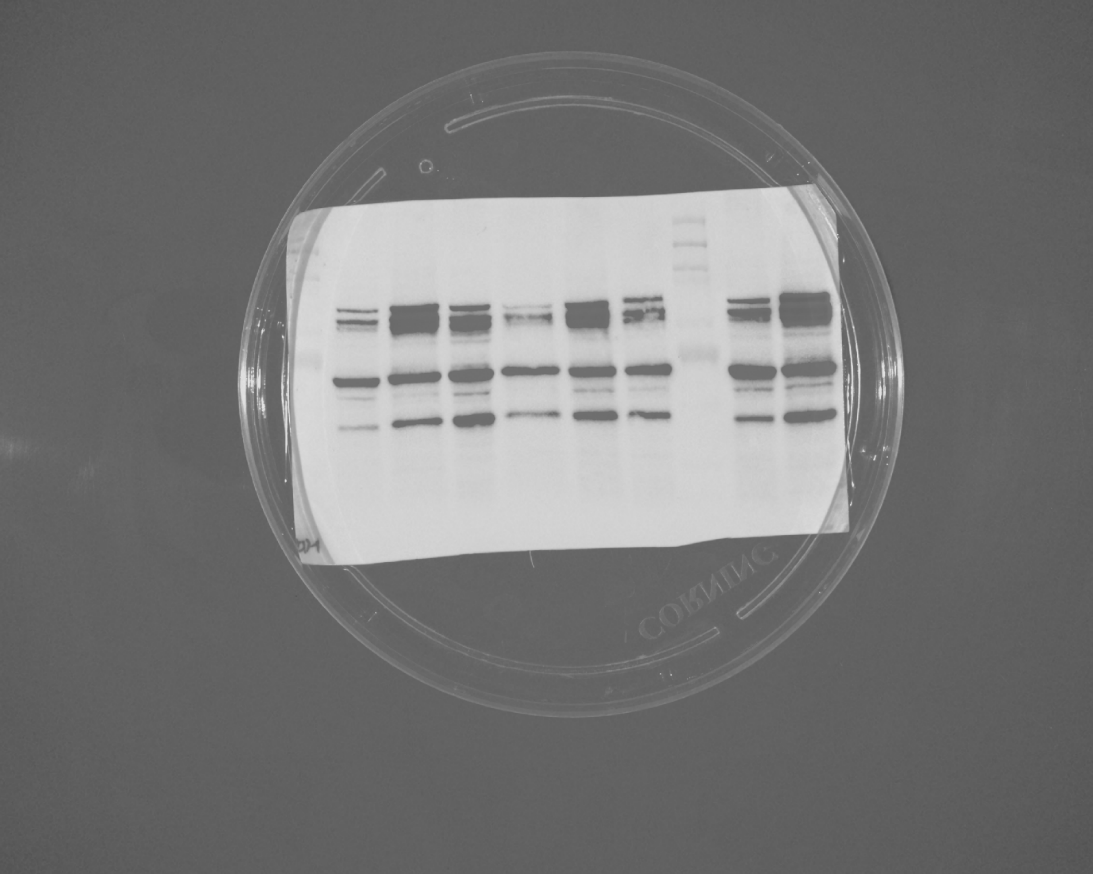


250

150

100

75

50

37

25

20

15

GAPDH

GLS1

Fig. 5f


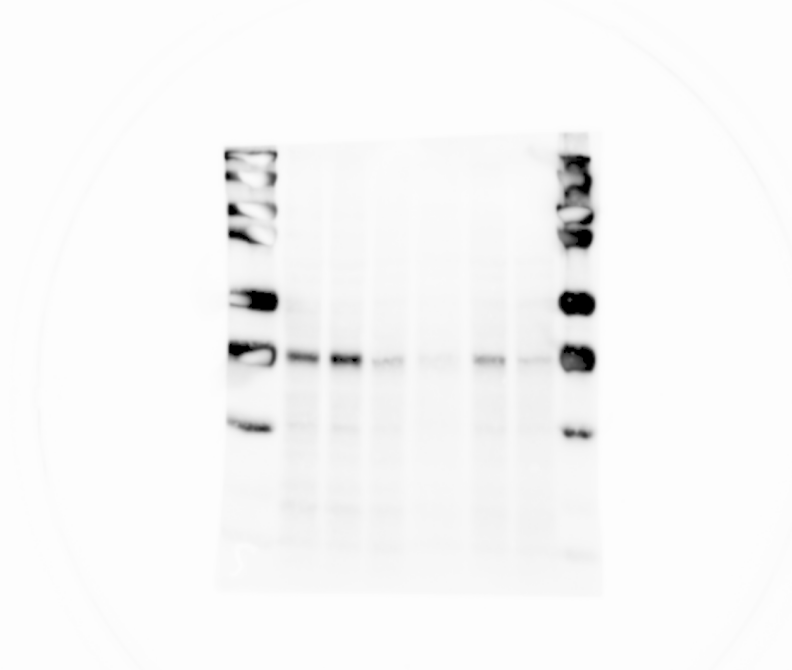

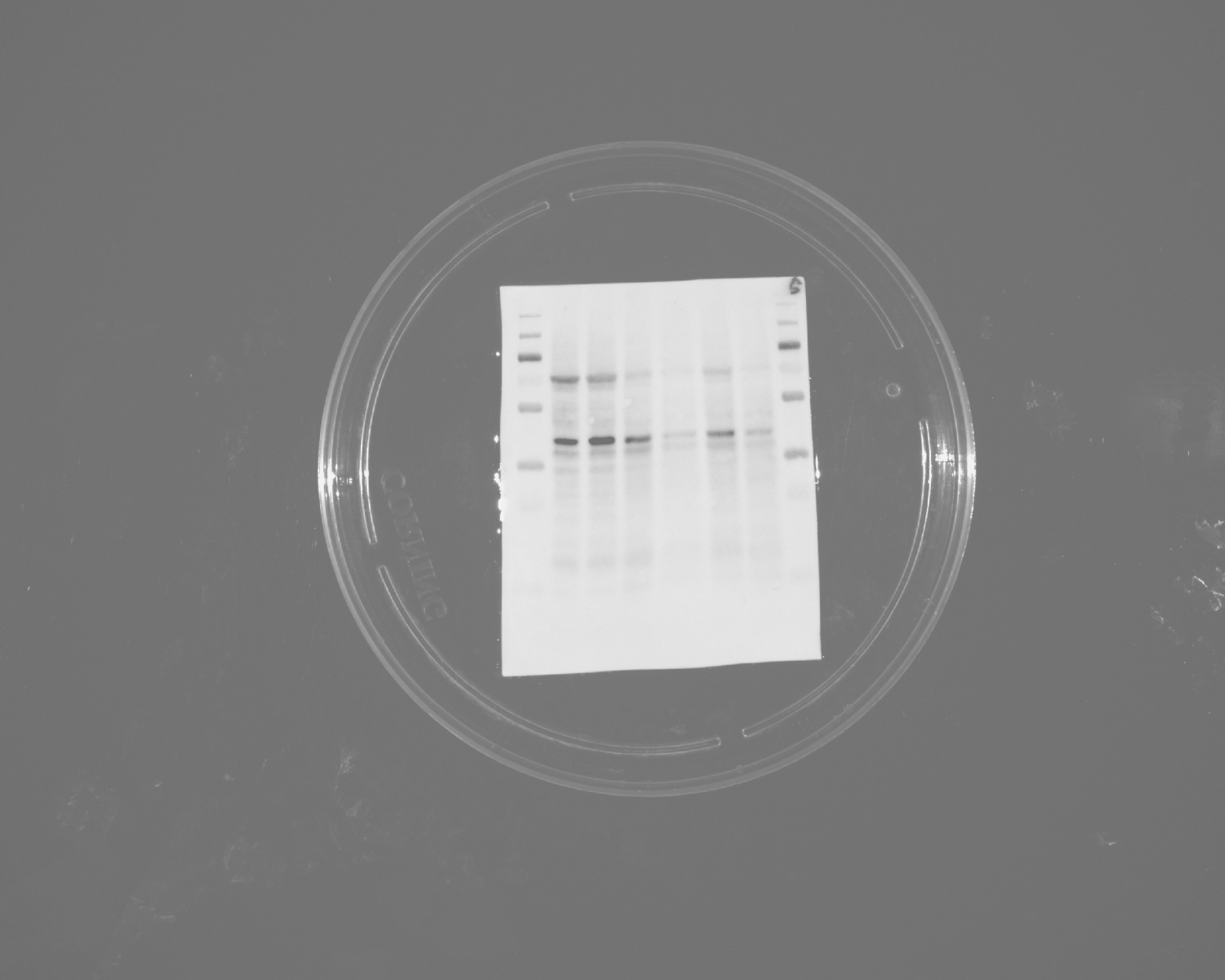

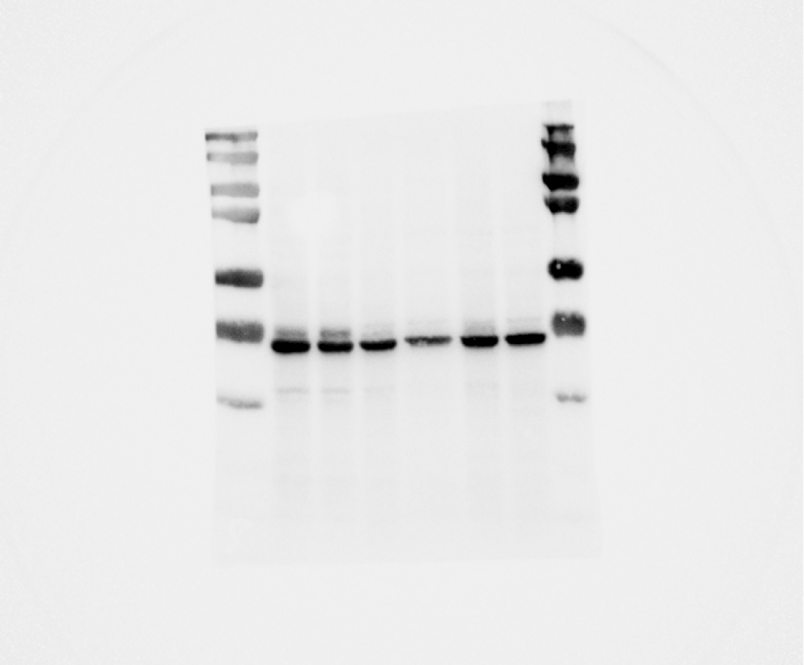


250

150

100

70

55

35

25

GAPDH

IKB

p-IKB

Fig. 5i


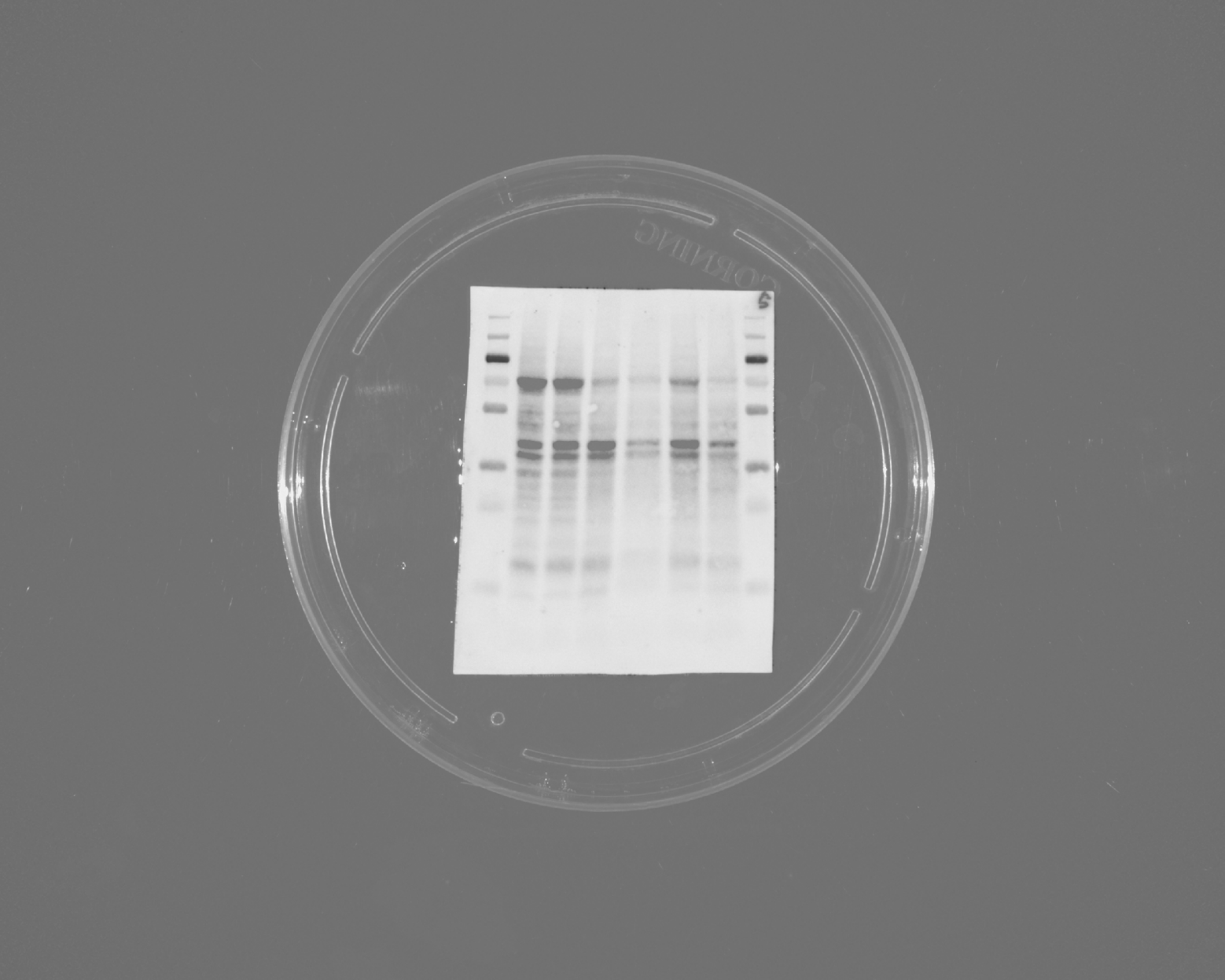

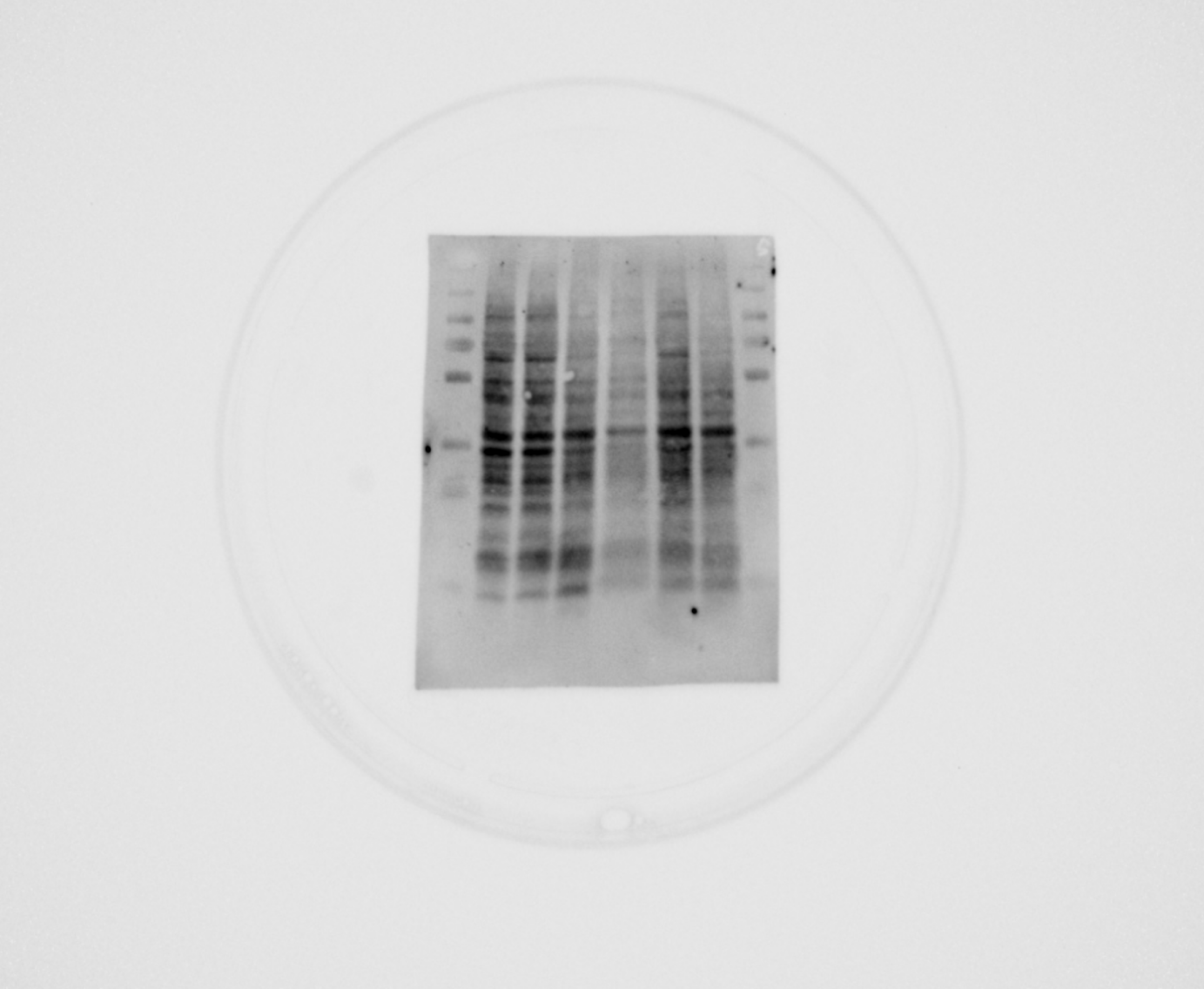

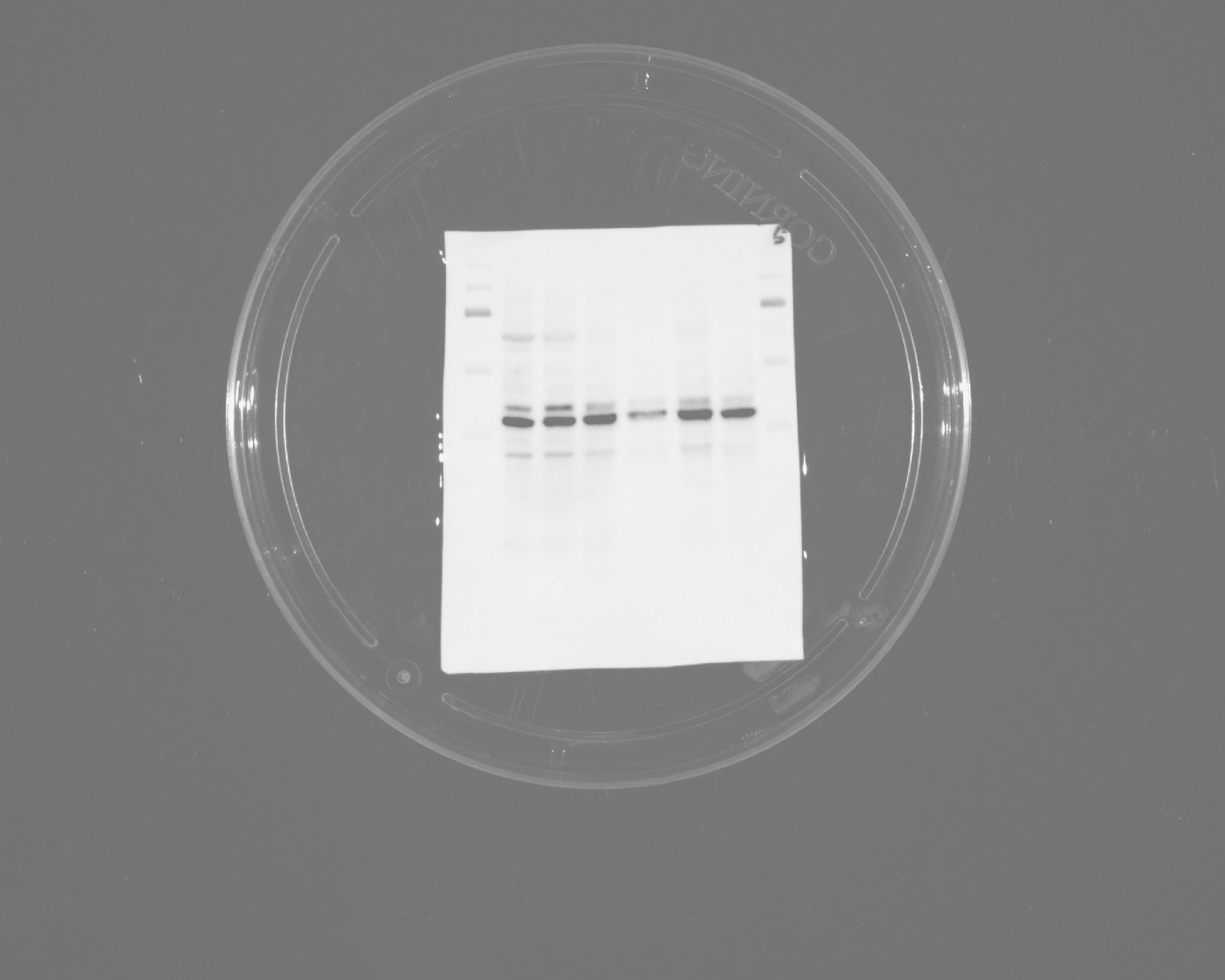


250

150

100

70

55

35

25

GAPDH

p-p65

p65

Fig. S4 Uncropped western blots
